# Movement-Related Differences Between Infants with High and Low Likelihood of Autism: A Longitudinal Study Using Markerless Motion Tracking

**DOI:** 10.3390/s26175643

**Published:** 2026-09-04

**Authors:** Mohammad Saber Sotoodeh, Georgina Donati, Ori Ossmy, Hannah Rowan, Gillian S. Forrester

**Affiliations:** 1 School of Psychology, University of Sussex, Brighton BN1 9RH, UK; h.j.rowan@sussex.ac.uk (H.R.); g.forrester@sussex.ac.uk (G.S.F.); 2 Departments of Experimental Psychology and Psychiatry, University of Oxford, Oxford OX1 2JD, UK; 3 Centre for Brain and Cognitive Development, School of Psychological Sciences, Birkbeck, University of London, London WC1E 7HX, UK; ori.ossmy@bbk.ac.uk; 4 Centre for Educational Neuroscience, School of Psychological Sciences, Birkbeck, University of London, London WC1E 7HX, UK

**Keywords:** autism spectrum disorder, infant development, kinematics, computer vision, longitudinal study, motor biomarkers, spontaneous movement

## Abstract

**Background:** Motor behaviour has been shown to differ in both complexity and variability between neurotypical and autistic populations. However, there is little research on the early development of specific kinematic features and their timing of emergence in early life. **Methods:** The current study examined the early development of kinematic features (duration, range, velocity, acceleration, entropy, and jerk) in infants with a high or low likelihood of autism using state-of-the-art automatic pose estimation models. We evaluated weekly home-recorded videos of naturalistic, spontaneous movements (controlling for clothing, background, and posture) from 60 participants (705 videos) longitudinally over the first six months of life. We automatically extracted kinematic data, including motion of the shoulders, elbows, hips, and knees. **Results:** Of the kinematic features examined, we found that the groups differed only in the variability of entropy, whereby the variability of entropy was lower in HL compared with LL infants across the first six months of infancy. **Conclusions:** These findings highlight the potential of non-invasive, home-based motion tracking to identify early divergences in infant motor trajectories among those with an elevated likelihood of autism.

## 1. Introduction

Autism is a neurodevelopmental condition currently diagnosed in early childhood [1]. The average age of diagnosis is often not until after the fifth year of life [2] because screening is largely predicated on delays and/or deficits in social and language skills [3]. Consequently, most autism studies to date have focused on social behaviours, as these form core diagnostic criteria later in life. Over the past two decades, research efforts have increasingly focused on identifying earlier markers of autism to enable timely intervention [4,5]. More recently, interest has turned to the early development of the motor system [6,7,8]. There is a growing recognition that early sensorimotor development forms the foundation for later, more complex cognition, and therefore differences or disturbances in the early sensorimotor system are likely to have cascading effects on later cognitive outcomes [9,10,11]. Yet the methodological challenges of evaluating motor trajectories in the first few months of life, particularly when differences are discernible only by diagnosis later in development, mean that we know very little about early differences in motor development. In this study, we used automatic pose estimation algorithms to calculate kinematic features from home-recorded videos of infants with a higher or lower likelihood of autism during the first six months of life.

### 1.1. Retrospective Methodologies and Early Motor Milestones

Multiple approaches have been taken to evaluate pre-diagnostic motor behaviour in those who later receive an autism diagnosis. One strategy has involved using retrospective data, such as home-recorded videos and parental reports. This means that, where an individual has received a diagnosis, they can refer to home-recorded videos or what parents can remember regarding the behaviour of a child when they were younger. However, these analyses have yielded conflicting results; for instance, while some studies found no motor differences in the first year [12], others reported significant delays in milestones such as postural control, sitting, and walking, emerging in the second and third years of life [13,14,15,16,17,18].

Evidence across motor assessment frameworks, including Eshkol–Wachman Movement Notation [19], the Alberta Infant Motor Scale (AIMS) [15], and WHO norms [20], remains inconsistent. Some data indicate significant differences in motor milestones [13,18], while other studies found no discernible differences between infants later diagnosed with autism and their neurotypical peers. Pioneering retrospective work in the late 1990s suggested that sensory–motor functions should be considered alongside social responses during infancy. However, subsequent replications using the Infant Motor Maturity and Atypicality Coding Scales (IMMACS) to analyse gross motor development—including prone/supine positions and crawling—reported no abnormalities that distinguished autistic infants from neurotypical controls, although differences were noted in developmentally delayed groups [16].

While retrospective approaches offer valuable access to early behaviour, they possess inherent limitations, most notably a lack of temporal alignment of data, precluding the assessment of true developmental trajectories. Retrospective studies are often reduced to “cross-sectional snapshots” that assume motor differences are stable. Given that development is inherently non-linear, compensatory mechanisms may mask differences at specific time points. Even studies attempting multi-point analysis often rely on serial cross-sectional comparisons rather than true longitudinal tracking [21,22,23].

Addressing these limitations of retrospective studies, prospective studies, which follow infants with a higher likelihood (HL) of autism due to familial recurrence, offer a robust alternative [24,25,26]. Autism is considered moderately heritable, with recurrence rates in siblings estimated at a range of 10–20% [27,28,29]. Crucially, membership in the HL group indicates an elevated probability rather than a confirmed diagnosis, as the majority of these infants will ultimately follow neurotypical developmental pathways. The prospective design allows for the comparison of developmental trajectories between HL and low-likelihood (LL) infants from early infancy until a diagnosis can be confirmed around 2–3 years of age [7].

However, prospective studies have tended to focus on social development or rely on generic developmental batteries like the Mullen Scales or Vineland for motor assessment [6,9,30,31]. These tools often lack the temporal resolution and sensitivity required to capture subtle kinematic atypicalities in the first months of life. For example, the Vineland’s motor items focus on later-emerging voluntary motor skills like crawling, sitting, and standing, etc, thereby missing the earliest developmental windows, comprising reflexive motor movement repertoires, where atypicalities may be most prominent.

### 1.2. General Movement Assessment (GMA) and Kinematic Analysis

The General Movement Assessment (GMA) provides a clinically sensitive alternative for evaluating involuntary spontaneous “general movements” (GMs) from birth to 20 weeks [32,33,34,35]. Typical GMs are characterised by variability in speed and amplitude, whereas atypical GMs are categorised as “poor repertoire,” lacking complexity [34,36]. Persistent poor repertoire is a robust predictor of various neurodevelopmental disorders, like cerebral palsy [23,37].

Retrospective evidence suggests that GMs may serve as early indicators of autism as well, with studies identifying frequent “poor repertoire” writhing movements or abnormal fidgety movements in infants later diagnosed as autistic [37]. Furthermore, repetitive limb movements have been identified as potential markers within the first year [38]. However, because the prognostic clinical scoring of the General Movement Assessment (GMA) was primarily designed to detect severe neurological damage like cerebral palsy, applying its classical criteria directly to autism is theoretically complex. In autism, early motor differences are potentially more related to subtle variations in motor control, adaptive behaviour, and restricted movement repertoires. Nevertheless, the foundational theory of GMs, which emphasises that typical infant movement is inherently complex and variable, remains highly relevant. Furthermore, the standardised GMA video-recording procedure (capturing spontaneous, unprompted supine movement) provides an ideal, ecologically valid framework for modern computer-vision techniques. By applying markerless pose estimation to these recordings, we can move beyond subjective clinical scoring and directly quantify continuous kinematic features—such as movement entropy and variability. This allows us to mathematically capture the complexity of spontaneous movement, providing objective metrics, not always available to the naked eye, that may better capture the distinct motor phenotypes associated with an elevated likelihood of autism.

Some recent studies have evaluated movement kinematics, including velocity, acceleration, jerk of motion, and variability in autistic individuals. Differences have been observed in HL infants [39], autistic children [40], and older autistic individuals [41,42,43] compared with LL and neurotypical individuals. With recent advancements in computer vision, researchers have begun applying kinematic methods used in older populations to quantitatively evaluate the early general movements in infants. For instance, Bruschetta, Caruso [44] analysed continuous movement features across infancy and reported atypical motor profiles in HL infants later diagnosed with autism. Doi, Iijima [45] showed that machine learning techniques could discriminate HL infants at 18 months based on their movement frequency, strength, count, ratio, and fluctuation. Bruschetta, Caruso [44] further demonstrated that markerless motion tracking could differentiate HL and LL infants as early as 10 days based on median velocity, area differing from the moving average, and periodicity. However, these studies rely on mean or median values of kinematic features [44,46]. Such aggregation may obscure important variability, which GM studies suggest is a critical indicator of difference [32,34,35,37]. Additionally, most kinematic studies remain lab-based, limiting ecological validity. Recent advances in remote data collection and computer vision now allow movement to be captured in infants’ natural home environments [47,48].

Although motor differences are not currently included in the autism diagnostic criteria [1], a growing body of evidence demonstrates their presence across the spectrum [49,50], fuelling interest in whether a distinctive “motor signature” exists [51]. Yet substantial challenges persist: recruiting infants early enough, collecting sufficiently frequent longitudinal data, and measuring motor features relevant to the earliest months of life.

### 1.3. The Present Study

The current study addresses existing challenges by longitudinally examining kinematic features in HL and LL infants across the first six months of life using weekly recorded videos in their home environment. Using state-of-the-art pose estimation, we analysed joint-based kinematic features, including duration, range, angular velocity, angular acceleration, entropy, and jerk, under controlled conditions. We tested a wide range of kinematic features based on the literature examining older children and adults. However, we predicted that: Drawing on infant GM literature, we predicted that (1) complexity (entropy), and movement variability would be the most informative measure, and (2) the shape of the developmental trajectories would differ significantly between the two groups. To test these predictions, we applied generalised linear mixed models (GLMMs) to examine the effects of both time and group on movement kinematics.

## 2. Materials and Methods

### 2.1. Participants

We recruited participants from across mainland UK and the USA by advertising on social media in the UK and using the SPARK (Simons Foundation Powering Autism Research) database in the USA. The inclusion criteria for HL babies were having parents or siblings with autism, or parents with a score equal to or higher than 14 on the Ritvo Autism Asperger’s Diagnostic Scale-14 [52]. Within the HL group, the infants recruited specifically via the RAADS-14 screener (*n* = 9) had a mean parental RAADS score of 26.1 (*SD* = 6.23), while those recruited via a confirmed family history (*n* = 20) of autism had a mean score of 21.9 (*SD* = 11.2). None of the participants in either the HL or LL groups were siblings of one another. A total of 60 participants (29 HL and 31 LL) who sent regular data were included in the analysis (for more information, see Supplementary File S1, Section S1). The participants who sent fewer than 4 videos were excluded from the analysis (Table 1). This study was approved by the ethical committee of the University of Sussex (reference: ER/GF235/1) in accordance with the ethical standards as laid down in the 1964 Declaration of Helsinki.

### 2.2. Procedure

Infant movement videos were recorded in the home, weekly, by parents/carers using a smartphone and a dedicated BabyGrow pack provided by the research team that included a phone holder, a yoga mat, and infant onesies in several sizes [48,53]. Parents/carers received a full instruction manual and Zoom tutorial on how to capture their infant’s motor behaviours. We asked parents/carers to reduce distractions from people or objects in proximity to the infant during video recordings. Parents/carers were instructed to use WeTransfer, a GDPR-compliant file-sharing service, to send their videos to the research team. To standardise the time used across videos, the most active 2 min were selected by a researcher blind to each infant’s group status of each video when the baby was awake, not fussy, and producing movements actively. In total, 705 videos were used for post-processing analysis.

### 2.3. Ritvo Autism and Asperger Diagnostic Scale-14

There is a possibility that adults, including biological parents in our study, have autism phenotypes which are not diagnosed. RAADS-14 is an established autism screening questionnaire and a shorter version of the Ritvo Autism and Asperger Diagnostic Scale-Revised (RAADS-R), used to measure autism characteristics in adults [52]. Scores range between 0 and 42. Eriksson, Andersen [52] reported a score of 14 or above reached a sensitivity for autistic traits with 97% accuracy and a specificity range of 46 to 64%, which is a promising measure in screening for autism in adults.

### 2.4. Kinematic Analysis

**Figure 1:**
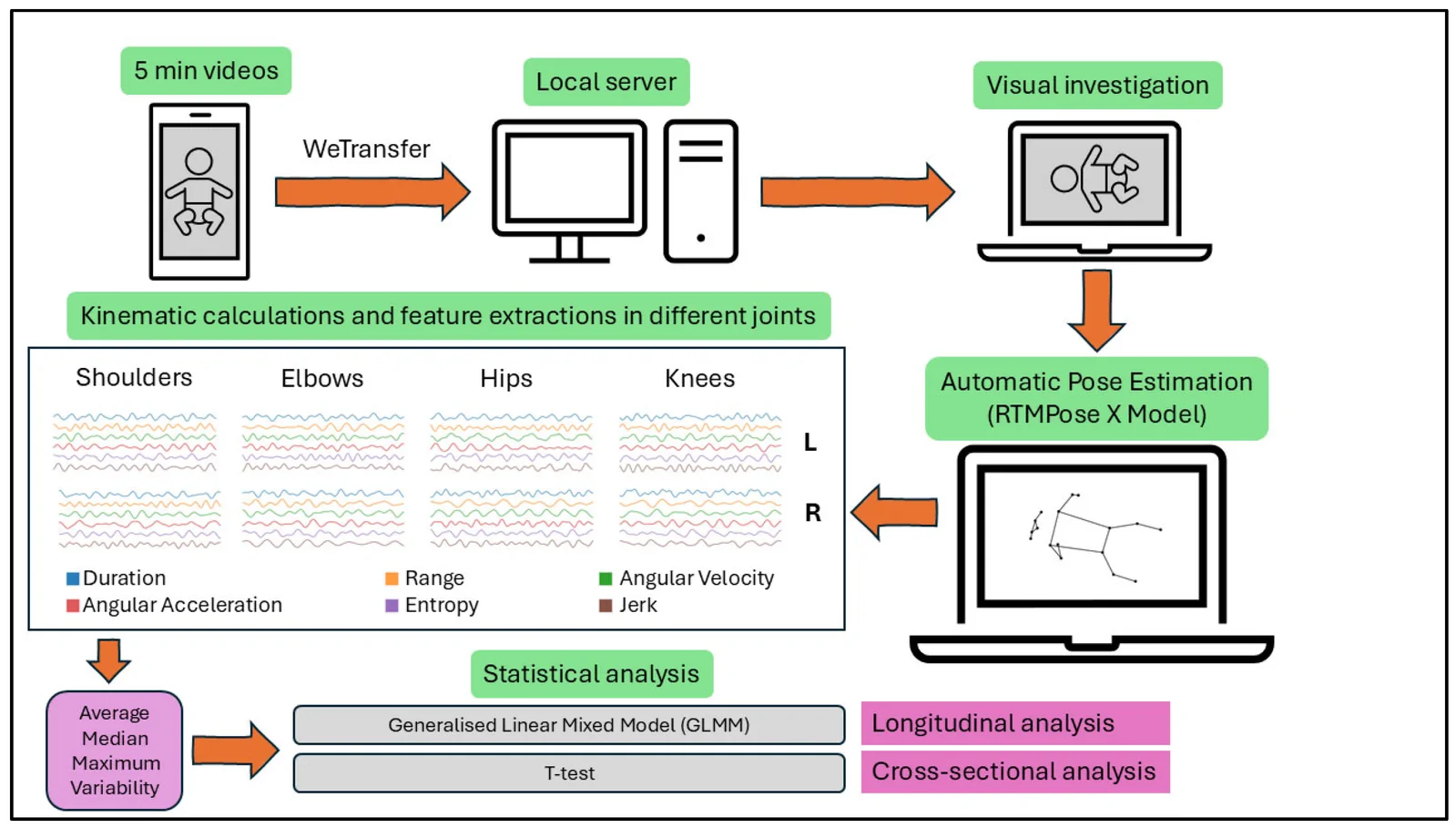
Schematic of the video recording, processing, feature extraction, and statistical analysis (starting from the top left).

A state-of-the-art RTMPose X Model [54] was employed to automatically extract the pose estimation of babies in all selected videos. A recent study showed that RTMPose is the most accurate model in infant studies [53]. It provided 17 body landmarks (in the x and y axes) for each frame of the video, including the nose and both the left and right eyes, ears, shoulders, elbows, wrists, hips, knees, and ankles, as a JSON file. For post-processing the data, we first recalculated all landmark locations based on the body’s centre to ensure that the changes in landmark location were not the result of camera displacement. Then, we smoothed the landmark positions in the X and Y axes using the Savitzky–Golay filter [55] using the SciPy package in Python 3.9, ensuring that complexity metrics like entropy reflect true biological movement patterns rather than algorithmic jitter. Crucially, visual inspection was not required to manually identify or segment individual discrete movements. Instead, we calculated the angles in the main joints: shoulders, elbows, hips, and knees. A single discrete movement was defined as the temporal window between two consecutive inflexion points (Figure 1). For example, increasing the angle in the elbow was defined as extension and decreasing it was defined as flexion (Supplementary Video S1). For each movement, we calculated kinematic features of duration, range [56], angular acceleration, angular velocity [42,44,57,58], entropy [59], and jerk [42,60,61] using the formulas in the Supplementary File S1 (Section S2) and choosing the peak for each movement time series.

### 2.5. Pose Estimation Quality

To ensure the fidelity of our kinematic features, we evaluated the tracking quality of the primary limb joints (shoulders, elbows, wrists, hips, knees, and ankles) across all included videos (N = 60 participants). The overall mean detection confidence score for these primary landmarks was 0.79. Landmark loss, defined as joint predictions falling below a 0.5 confidence threshold, was minimal at 1.01%, and these rare gaps were handled via linear interpolation. Importantly, Welch’s independent *t*-tests confirmed there were no systematic differences between the HL and LL groups in either mean confidence (HL = 0.78, LL = 0.79, *p* = 0.83) or landmark loss percentage (HL = 1.12%, LL = 0.89%, *p* = 0.57).

### 2.6. Kinematic Entropy Calculation and Interpretation

Given its central role in our hypothesis, the conceptual calculation of kinematic entropy warrants specific description (full mathematical formulations for all metrics are available in the Supplementary File S1, Section S2). We utilised Shannon Joint Entropy to quantify the complexity of each discrete movement event because entropy is the scientific measure of disorder or randomness and would be expected to be increasing in a more complex system. For each identified flexion or extension phase, the continuous time-series data for joint angle, angular velocity, and angular acceleration were stacked to represent a three-dimensional kinematic state space. We computed the joint probability distribution of these variables using 10 discrete bins. Entropy was then calculated directly from this distribution. In the context of infant motor development, this entropy value serves as a quantitative measure of movement complexity: higher entropy reflects highly complex, unpredictable, and fluent movement repertoires typical of healthy neurodevelopment, whereas lower entropy indicates highly predictable, repetitive, or monotonous movement patterns.

### 2.7. Kinematic Feature Aggregation

Mathematical formulations for all extracted features (including angular velocity, acceleration, jerk, and entropy) are detailed in Supplementary File S1 (Section S2). Kinematic variables were calculated for each discrete movement event across all primary joints (shoulders, elbows, hips, and knees). To capture a holistic representation of the infant’s motor repertoire, these individual joint movements were pooled to create a whole-body summary.

Due to the high volume of spontaneous movements recorded during each 2 min session, we temporally aggregated these discrete events into session-level (age-level) data points for each participant. To evaluate the central tendency and temporal dynamics of the motor repertoire, we calculated the mean, median, maximum, and variability across all extracted movements within a given session. To measure the temporal fluctuation of these movements, we utilised the Coefficient of Variation (CV=σ/μ) as our primary variability index. These session-level aggregated metrics served as the final continuous variables utilised in our longitudinal statistical models.

### 2.8. Statistical Analysis

For statistical analysis, we used glmmTMB [62] and lme4 [63] packages in R on kinematic measures (Supplementary File S1 Section S3 for descriptive data). A generalised linear mixed model (GLMM) was fitted to examine the effects of group (prediction I) and age (prediction II) on all movement features, with age (week) and group and their interaction as predictors and participant as a random effect. Furthermore, since the gestational age was different between the two groups, it was added to the model as a covariate. Given the presence of only positive values, a Gamma distribution with a log link was used. To capture the hypothesised non-linear trajectory of early motor development, we considered both linear and quadratic effects for age in weeks.

To rigorously control the false discovery rate (FDR) while preserving statistical power for our specific hypotheses, we applied a tiered multiple-comparison correction framework. Based on our a priori prediction that entropy and variability would be the most informative discriminators, variables were divided into two analytic families:

Primary Confirmatory Analysis: GLMMs evaluating entropy and variability metrics.

Secondary Exploratory Analysis: GLMMs evaluating all remaining kinematic parameters (e.g., duration, range, angular velocity, angular acceleration, and jerk).

The Benjamini–Hochberg (FDR) procedure was applied independently within these respective families to adjust the *p*-values for the fixed effects. Statistical significance was defined as an FDR-adjusted *p* < 0.05. Finally, to determine specific developmental time points where the kinematic trajectories significantly diverged between the high and low likelihood groups, we conducted model-based marginal contrasts. Rather than performing independent cross-sectional tests, we extracted the predicted group differences at each recorded week directly from the fitted GLMMs. This was achieved by multiplying the group-contrast design matrix by the fixed-effect coefficients, with standard errors calculated via the model’s conditional variance–covariance matrix. This approach ensures that point-by-point comparisons properly account for the overall non-linear trajectory, participant-level random effects, and covariates (e.g., gestational age). The resulting Z-statistics for the weekly group gaps were evaluated for significance, and *p*-values were adjusted using the Benjamini–Hochberg False Discovery Rate (FDR) procedure to strictly control the family-wise error rate across the developmental timeline [64].

## 3. Results

To rigorously control for multiple comparisons, all *p*-values reported in this section have been adjusted using the Benjamini-Hochberg (FDR) procedure unless otherwise noted. To evaluate the main effect of group, a GLMM was performed and we observed a significant main effect of group only for entropy variability, β = 0.0581, SE = 0.02, z = 2.82, *p* = 0.033, 95% CI [0.017, 0.098], e^β^ = 1.06 (see Supplementary File S1, Section S4 for all GLMM results), showing that the LL group had higher entropy variability than the HL group.

To evaluate developmental trajectories, we first looked at the main effect of time (linear and quadratic) and its interaction with group.

**Figure 2:**
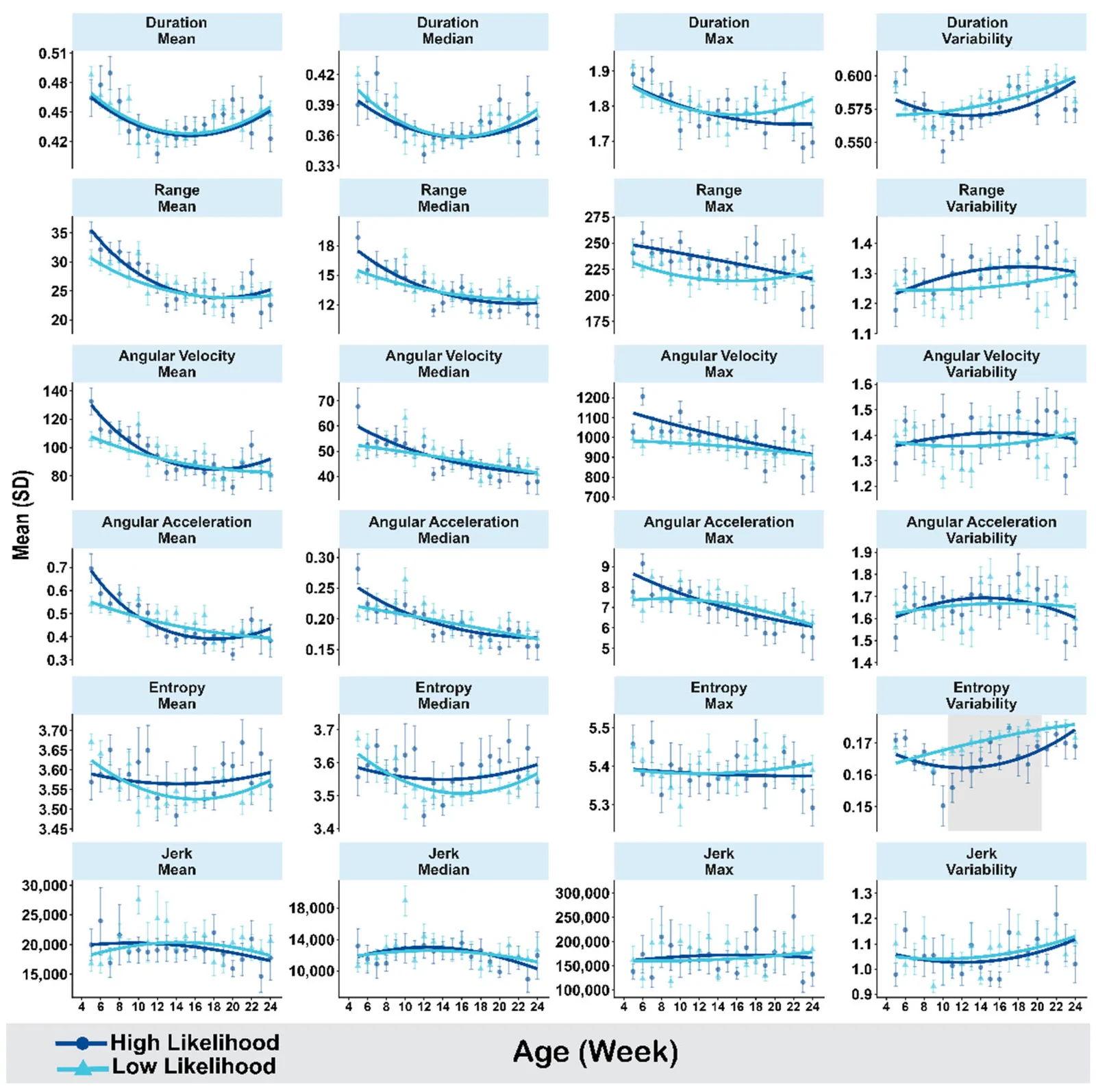
Developmental trajectories of kinematic measures by group likelihood. Developmental changes in kinematic measures (mean, median, maximum, and variability) across the first 24 weeks for the high likelihood (HL; dark blue) and low likelihood (LL; light blue) groups, where the trajectories are the GLMM predictions (see Supplementary File S1, Section S4). Most kinematic magnitudes (range, angular velocity, and angular acceleration) decrease significantly over time, while duration shows a characteristic U-shaped trajectory. Group differences are most prominent in entropy variability, where the HL group shows a lower value than the LL group. Looking at the weekly level comparisons (FDR-adjusted model-based marginal contrast), there were only significant differences between HL and LL groups in entropy variability between weeks 11 and 21 (*p* < 0.05).

As Figure 2 reveals, duration shows a quadratic effect of age in mean, median, and variability measures (*p* < 0.005), and a linear effect for max (*p* < 0.005). Range shows a quadratic effect of age in mean (*p* < 0.005) and a linear effect of age for mean and max (*p* < 0.005). Angular velocity and angular acceleration show a quadratic effect of age for mean (*p* < 0.005) and a linear effect of age for mean, median, and max (*p* < 0.005). Entropy shows a quadratic and linear effect of age for variability (*p* < 0.005), and jerk shows a quadratic and linear effect of age for median (*p* < 0.005).

Furthermore, the interaction of group × age (quadratic) was significant for entropy variability, β = −0.0007, SE = 0.0002, z = −3.65, *p* = 0.002, 95% CI [−0.001, −0.0003], e^β^ = 0.999, where for entropy variability, the HL group showed a quadratic trajectory, decreasing the variability in the first three months and rapidly increasing from the fourth month, and the LL group showed a linear increasing trajectory from the early weeks. Marginal contrasts (Supplementary File S1, Section S5) indicated that entropy variability was significantly lower in the high likelihood group specifically between weeks 11 and 21 (*p* < 0.05). To ensure these results were not driven by week-to-week sample size imbalances, a sensitivity analysis utilising a random slope for age was conducted, confirming the quadratic group × age interaction remained highly significant (*p* < 0.001; see Supplementary File S1, Section S6).

Also, the interaction of group × age (quadratic) was significant for angular acceleration mean, β = −0.0027, SE = 0.0009, z = −2.83, *p* = 0.018, 95% CI [−0.0045, −0.0008], e^β^ = 0.997, where the HL group showed a sharp decrease in the first 12 weeks and a slow increase after week 20, producing a U-shaped trajectory, while the LL group showed a linear decrease in angular acceleration.

## 4. Discussion

This study aimed to investigate the development of kinematic features of infants’ spontaneous movements with a high and low likelihood of autism. Kinematic features (duration, range, angular velocity, angular acceleration, entropy, and jerk) were assessed longitudinally from week 4 to 24. We specifically examined the mean, median, maximum, and variability of these kinematic features to explore which factors discriminate between infants with a high and low likelihood of autism. Our findings indicated that (I) there was a statistically significant difference between groups in the variability of entropy, and (II) there was considerable variation in the trajectory of kinematic features, with some features changing in a linear and some in a quadratic course.

### 4.1. Variability of Complexity Discriminates HL from LL Infants

Recent technological advancements have provided the opportunity to study infants’ movements at high-frequency scales. However, if researchers are unable to select the most relevant features and measures, it becomes challenging to analyse the data effectively and identify meaningful differences. The mean and median are two of the most common candidates for these types of analyses. In the current study, the mean, median, and maximum of entropy did not show significant differences between HL and LL groups, which is inconsistent with the GM literature indicating higher movement complexity in typically developing infants [33,34,35,37]. The variability of entropy was a discriminative factor, which is consistent with the literature on GMs, showing higher variability as an index of neurotypicality [33,34,65].

In a recent study, Bruschetta, Caruso [44] demonstrated that movement velocity could discriminate between HL and LL groups only when infants were ten days old, but not at 6, 12, 18, 20, and 24 weeks. A careful examination of their data suggests that the number of participants in the first session (10 days after birth) was smaller than in subsequent sessions, which might explain this observed difference. The uneven number of participants in different sessions is not uncommon in longitudinal studies; however, it challenges the interpretation of the findings in the cross-sectional analysis. We encountered similar challenges in our analysis; although we aimed to analyse infants’ movements from birth, most of the data for our HL infants were submitted by the end of the first month. To mitigate bias due to a lack of participants in the first month, we excluded those initial sessions from our analysis. The inconsistency in the number of participants at different time points in longitudinal studies raises concerns about later cross-sectional statistical analysis and highlights the need for a longitudinal comparison approach.

### 4.2. Early Developmental Trajectories

Analysis of the various kinematic features showed significant developmental changes in duration, range, angular velocity, and angular acceleration during the early months of life. Moreover, these kinematic features are drawn from motor behaviour in the infant’s own home, providing an ecologically relevant, naturalistic setting that is often absent in lab-based studies. The observed mixed developmental patterns, dominated by quadratic trajectories, align with the transition to fidgety movements (FMs). This period, which typically occurs between 3 and 5 months, is characterised by movements of smaller amplitude and lower speed [34]. Our data quantify this transition through the rapid decreases in duration, range, and angular velocity observed in the first three months. Crucially, while both groups followed similar developmental timelines in most of the kinematic measures, the HL group exhibited a distinct quality of transition, characterised by significantly lower variability of entropy. This suggests that while the biological clock for motor milestones may remain intact in the HL group, the refinement of movement complexity is uniquely impacted.

As reflexive behaviours are progressively replaced by voluntary, goal-directed movements after three months, our findings provide a high-resolution map of this functional shift. For example, the U-shaped trajectory of movement duration may reflect the infant’s transition from rapid, involuntary reflexes to the longer, more deliberate durations required for early reaching and environmental exploration. The “valuable sensitivity” of these features lies in their ability to objectively quantify the onset of motor complexity (entropy), possibly providing a digital signature of neurodevelopmental conditions that precedes the visible delay of gross motor milestones. Increased muscle strength enables the infant to overcome gravity, and the utilisation of environmental information promotes an increase in varied, goal-oriented movements [66]. These changes are associated with a transition from subcortical to cortical motor processing [67].

Our results also showed that variability increased in entropy between all kinematic features during the early months. The Group × quadratic Age interaction for entropy variability indicated different trajectories: variability decreased in the HL group during the first three months and then increased rapidly, whereas it increased linearly in the LL group. This developmental pattern supports the GM literature that considers the increasing variability of movement over time [32,33,35,65,68,69]. The quadratic developmental trajectory of movement has been reported in previous studies [70,71,72]. Kinoshita, Furui [71], in their longitudinal study using markerless video analysis of infants from 1 to 15 weeks, demonstrated the trajectory of their movements through U-shaped and inverted U-shaped changes in various kinematic measurements. According to a dynamic systems approach to motor control, spontaneous movements arise from the dynamic interaction among neural, musculoskeletal, and environmental factors [73], and the development of motor behaviours can be characterised as a convergence to stable attractors [74]. Conversely, the fundamental mechanism of development involves a decrease in redundancy, based on the premise that redundant neurons and synapses are initially produced, with only the relevant ones subsequently selected through neuronal death and synaptic elimination to form an organised network [75,76]. Based on observations of motor development, Bernstein claimed that the number of degrees of freedom of movement increases as the performance of behaviours improves [72,77]. By combining these two distinct perspectives on development, it is plausible that both an increase and a decrease in degrees of freedom occur concurrently. From a neural mechanism standpoint, the observed U-shaped complexity (variability of entropy) of movement patterns in the HL group suggests that the cortex is involved in both the generation of complex motor patterns and the transformation of general movement (GM) patterns to voluntary movements during development. Provided that the circuits responsible for generating rhythmic movements are not directly affected, they can produce movement. However, the quality of these movements is likely modulated by corticospinal tracts and can therefore be affected by changes within these structures [78].

Despite its novel contributions, this study has several limitations. First, our sample comprised infants identified as HL based on family history rather than confirmed diagnostic outcome; a longitudinal follow-up is required to determine which HL participants ultimately receive an autism diagnosis. Second, broader demographic representation (including varied ethnicity and cultural backgrounds) is needed before generalising these findings. Finally, although we provided comprehensive instructions to parents, home-recorded video quality and recording conditions varied between families, which, despite our selection criteria, could have influenced pose estimation accuracy and subsequent kinematic measurements.

Building on these results, future research should assess clinical predictive validity by tracking HL infants to at least a diagnostic age to establish which kinematic markers (e.g., variability of entropy) are most strongly associated with later autism diagnoses. Future studies should also include infants from a range of neurodevelopmental profiles (e.g., other developmental disorders) to test the specificity of observed motor patterns. Furthermore, research could explore whether the early identification of motor differences enables timely motor-based supports, and assess their impact on subsequent social, communicative, and cognitive outcomes. Determining optimal developmental windows by linking kinematic trajectories to critical periods of neuroplasticity will also be vital. By addressing these limitations and pursuing these avenues, future studies can refine early motor indicators associated with autism, enhance automated assessment tools, and ultimately support earlier, more personalised intervention strategies.

## 5. Conclusions

This longitudinal study demonstrates that high-resolution kinematic analysis revealed specific movement features, particularly variability of entropy, which successfully differentiated between HL and LL groups longitudinally in the first six months. These findings suggest that complex, dynamic measures of movement features may capture subtle neurodevelopmental divergences well before they become apparent in standardised assessments.

The non-linear trajectories observed across kinematic variables underscore the evolving nature of infant motor control, reflecting the transition from spontaneous to goal-directed movements and the maturation of underlying neural circuits. Crucially, the choice of summary statistic markedly influenced the detection of group differences, highlighting the importance of feature selection in automated movement analysis. By emphasising metrics that capture variability, researchers and clinicians can gain a more nuanced understanding of early motor development.

## Figures and Tables

**Table 1:** Demographic information of participants.

Variable		HL (29)	LL (31)	Test Index	*p*
Gender	Boy	16	17	0.0 *	1.0
	Girl	13	14		
Gestational age (weeks)		38.928 ± 1.741	40.193 ± 1.194	−3.219 ^!^	0.002
Birth weight (kg)		3.577 ± 0.744	3.503 ± 0.451	0.456 ^!^	0.65
Mother’s age (years)		34.482 ± 3.112	33.62 ± 3.509	0.989 ^!^	0.326
Father’s age (years)		36.423 ± 3.931	37.034 ± 5.84	−0.459 ^!^	0.647
RAADS-14		23.346 ± 9.87	4.931 ± 3.4	7.867 ^!^	<0.001

* Chi-square test. ^!^ *t*-test.

## Data Availability

The datasets used and/or analysed during the current study are available from the corresponding authors on reasonable request.

## Supplementary Materials

The following supporting information can be downloaded at: https://www.mdpi.com/article/10.3390/s26175643/s1, Section S1: Participants’ further information; Section S2: Kinematic calculations and formulas; Section S3: Descriptive findings; Section S4: GLMM results; Section S5: Marginal contrasts; Section S6: Sensitivity Analysis; Video S1: Right Elbow Detections.
