# Supplementary material for: Movement-Related Differences Between Infants with High and Low Likelihood of Autism: A Longitudinal Study Using Markerless Motion Tracking
Source: Sensors (Basel). 2026 Sep 4;26(17):5643. doi: 10.3390/s26175643 (PMC13568383; doi:10.3390/s26175643)
Supplement: Supplementary file 1 [file sensors-26-05643-s001.zip › Supplementary Materials.pdf]

S 1

Participants’ further information

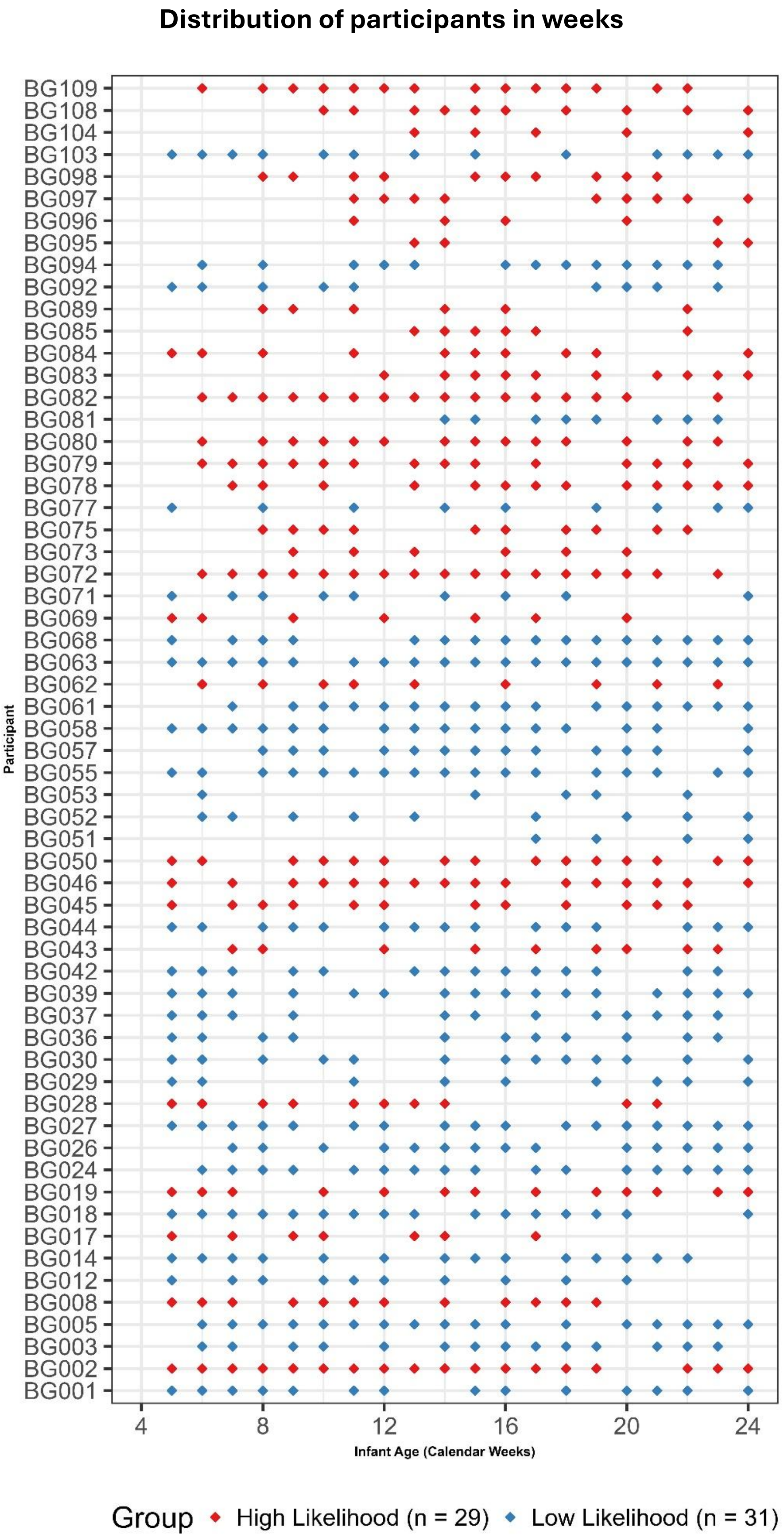
Distribution of participants in weeks
Participant
BG109
BG108
BG104
BG103
BG098
BG097
BG096
BG095
BG094
BG092
BG089
BG085
BG084
BG083
BG082
BG081
BG080
BG079
BG078
BG077
BG075
BG073
BG072
BG071
BG069
BG068
BG063
BG062
BG061
BG058
BG057
BG055
BG053
BG052
BG051
BG050
BG046
BG045
BG044
BG043
BG042
BG039
BG037
BG036
BG030
BG029
BG028
BG027
BG026
BG024
BG019
BG018
BG017
BG014
BG012
BG008
BG005
BG003
BG002
BG001
4
8
12
16
20
24
Infant Age (Calendar Weeks)
Group
High Likelihood (n = 29)
Low Likelihood (n = 31)

Presence of participants in weeks

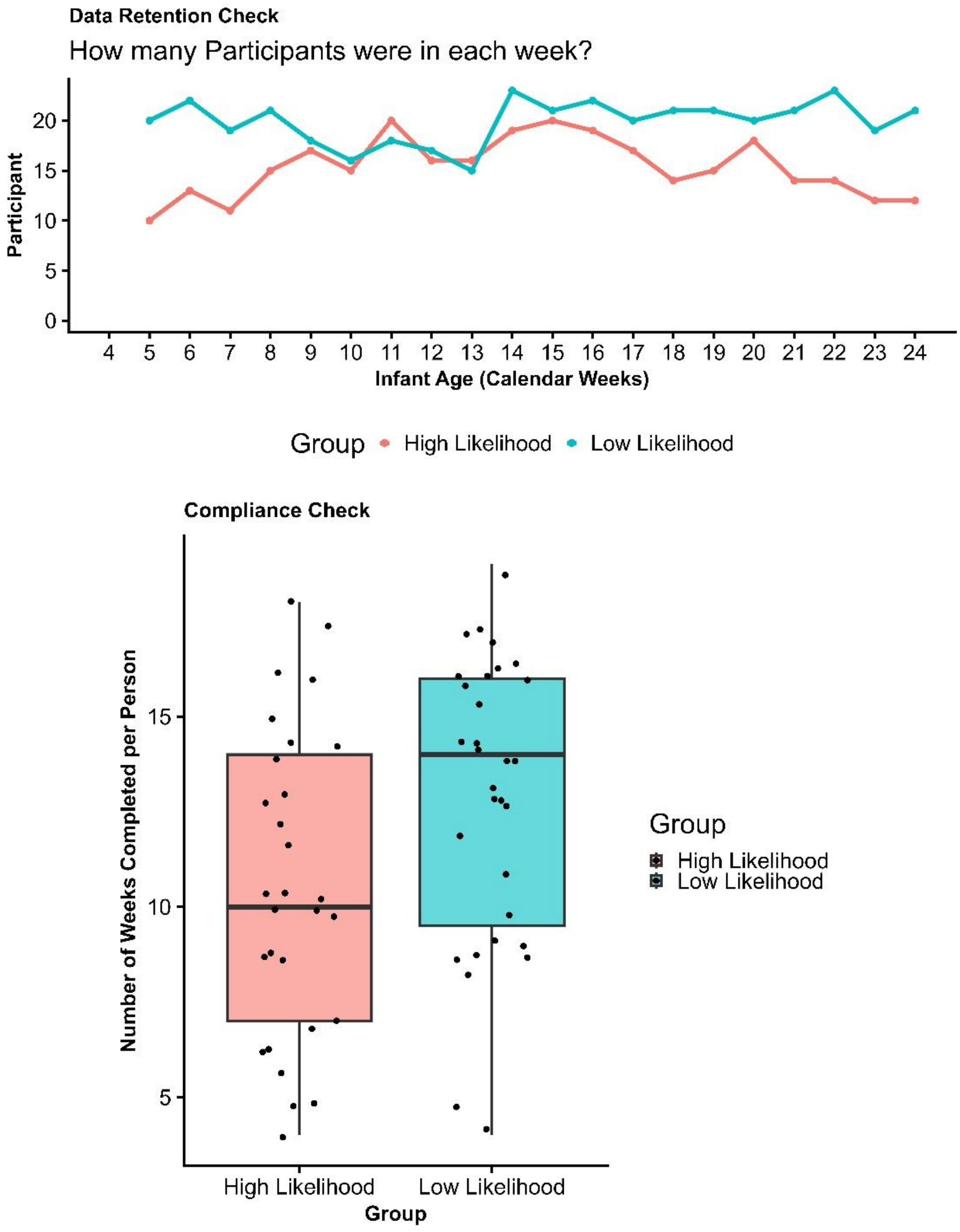

Table S1. Further descriptive information regarding the participants' presence in the studied weeks.

Group	Total Participants	Total Observations	Average Observation Per Person	Minimum Observation Per Person	Maximum Observation Per Person
High Likelihood	29	307	10.6	4	18
Low Likelihood	31	398	12.8	4	19

S 2

Kinematic calculations and formulas

Duration

To calculate the duration of each movement, we used the following formula, in which d is the duration of the movement, $e_{i,t}$ is the ending time, and $s_{i,t}$ is the starting time [57].

$$d = e_{i,t} - s_{i,t}$$

Range

To calculate the range of movement, we used the following formula, in which r is the calculated range, $e_{i,a}$ is the endpoint joint angle, and $s_{i,a}$ is the starting point of the joint angle [56].

$$r = e_{i,a} - s_{i,a}$$

Angular Velocity

In this formula, ω_i represents the angular velocity at the *i*-th time point, Θ_i is the angle recorded at the *i*-th time point, and t_i denotes the corresponding time value [58]. To prevent division by zero, any zero values in the time differences (Δt) were replaced with *NaN* (not a number). To reduce noise amplification, the resulting signal was smoothed using a Savitzky–Golay filter.

$$\omega = SavGol\left(\frac{\theta_{i+1} - \theta_i}{t_{i+1} - t_i}\right)$$

$$\omega_i = Angular\ velocity\ at\ time\ step\ i$$

$$\theta_i = Angle\ at\ time\ step\ i$$

$$t_i = Time\ at\ time\ step\ i$$

Angular Acceleration

In this formula, a_i represents the angular acceleration at the *i*-th time point, ωi is the angular velocity at the *i*-th time point, and t_{i+1} and t_{i+2} denote the corresponding time values. The scaling factor (×10^{-3}) was applied to ensure unit consistency [57].

$$a_i = SavGol\left(\frac{\omega_{i+1} - \omega_i}{t_{i+1} - t_i}\right) \times 10^{-3}$$

$$a_i = Angular\ acceleration\ at\ time\ step\ i$$

$$\omega_i = Angular\ velocity\ at\ time\ step\ i$$

$$t_{i+1}\ and\ t_i = Corresponding\ time\ points$$

Entropy

Entropy was calculated to capture the variability and unpredictability of the data [59]. A histogram of the selected feature was first constructed using an automatic binning strategy. The Shannon Entropy (in bits) was then calculated as follows:

$$H = -\sum_{i=1}^{n} p_i log\ _2(p_i)$$

$$H = entropy(bits)$$

$$p_i = the\ probability\ density\ for\ the\ i - th\ bin$$

$$n = total\ number\ of\ bins$$

Higher entropy values reflect greater variability, while lower values indicate more predictable patterns.

Jerk

Jerk was calculated as the rate of change in acceleration, providing insight into movement smoothness. Velocity and acceleration were derived via discrete differentiation. The final jerk magnitude was smoothed using a Savitzky–Golay filter [61]. Finally, the jerk was derived as follows:

$$J = SavGol\left(\sqrt{\left(\frac{da_x}{dt}\right)^2 + \left(\frac{da_y}{dt}\right)^2}\right)$$

$$J = jerk\ magnitude$$

$$da_x\ and\ da_y = acceleration\ in\ the\ x - and\ y - directions$$

$$t = time$$

S 3

Descriptive findings

Table S2. Descriptive statistics for the kinematic variabilities (average).

Variable	Duration		Range		Angular Velocity		Angular Acceleration		Entropy		Jerk	
	Mean	SD	Mean	SD	Mean	SD	Mean	SD	Mean	SD	Mean	SD
Week 5	0.464	0.058	37.252	7.99	132.559	29.641	0.695	0.2	3.569	0.142	50277.037	96090.198
	0.5	0.051	30.712	6.076	105.976	23.837	0.541	0.151	3.671	0.088	16761.92	5725.134
Week 6	0.478	0.069	32.114	8.052	112.713	36.242	0.588	0.23	3.588	0.159	23980.926	20433.983
	0.482	0.072	29.546	5.247	104.557	22.768	0.539	0.177	3.641	0.151	16688.719	6991.868
Week 7	0.49	0.056	31.796	5.237	111.076	22.458	0.543	0.127	3.651	0.126	16913.809	8300.455
	0.459	0.06	31.69	11.492	115.263	42.958	0.596	0.242	3.586	0.146	32208.516	61639.221
Week 8	0.461	0.05	33.352	9.163	117.396	33.259	0.585	0.155	3.574	0.111	30784.092	39764.113
	0.443	0.067	31.433	9.033	115.569	40.084	0.608	0.302	3.555	0.152	21325.697	9625.646
Week 9	0.443	0.072	29.598	8.625	106.471	35.063	0.508	0.168	3.589	0.265	24017.122	22676.365
	0.464	0.058	27.302	6.63	97.695	26.329	0.499	0.163	3.615	0.126	19094.006	7901.234
Week 10	0.434	0.054	30.34	8.335	110.784	31.036	0.55	0.182	3.662	0.344	18974.098	8022.135
	0.418	0.056	31.477	7.728	116.305	32.899	0.615	0.275	3.493	0.135	27563.535	9324.202
Week 11	0.424	0.032	28.768	6.64	103.991	26.374	0.523	0.186	3.638	0.273	18871.288	6097.127
	0.427	0.038	24.463	8.058	87.595	31.884	0.451	0.21	3.531	0.099	20412.791	13815.497
Week 12	0.407	0.034	26.005	5.433	94.375	22.745	0.442	0.144	3.528	0.226	19080.352	7897.895
	0.421	0.042	27.885	6.256	101.495	22.661	0.506	0.139	3.505	0.099	24357.436	18393.671
Week 13	0.428	0.051	23.795	6.842	87.82	29.468	0.429	0.166	3.609	0.28	19709.524	8394.519
	0.426	0.039	26.128	6.83	94.318	26.383	0.468	0.165	3.514	0.1	23918.557	12028.361
Week 14	0.423	0.038	24.618	8.882	88.486	34.517	0.406	0.158	3.533	0.229	19783.462	8406.005
	0.428	0.053	26.4	5.694	95.312	23.784	0.502	0.144	3.546	0.21	19401.074	8309.771
Week 15	0.435	0.05	24.935	6.373	87.802	25.647	0.413	0.143	3.562	0.168	19504.93	7677.251
	0.423	0.037	23.859	6.357	85.873	26.199	0.426	0.159	3.513	0.096	21410.268	10305.258
Week 16	0.428	0.061	25.477	9.015	91.505	35.791	0.419	0.18	3.59	0.24	21211.263	9965.037
	0.431	0.047	24.727	8.16	90.108	34.215	0.46	0.198	3.521	0.113	20245.054	11953.705
Week 17	0.437	0.032	23.119	8.893	82.373	36.297	0.372	0.182	3.525	0.094	19092.94	9301.432
	0.432	0.026	26.542	7.22	96.179	30.927	0.493	0.192	3.526	0.068	21599.348	11150.38
Week 18	0.443	0.055	26.562	7.678	93.292	31.032	0.43	0.147	3.651	0.332	22787.147	10637.36
	0.453	0.066	25.507	16.032	89.317	60.24	0.436	0.323	3.57	0.163	33750.369	57104.168
Week 19	0.448	0.035	22.462	8.121	78.297	32.903	0.383	0.184	3.712	0.316	18445.225	8219.23
	0.454	0.041	22.404	5.785	77.74	22.33	0.38	0.125	3.579	0.098	16625.209	6240.853
Week 20	0.47	0.064	20.717	5.359	71.283	20.323	0.318	0.103	3.623	0.149	15557.245	5800.371
	0.453	0.061	24.165	7.28	85.378	28.489	0.41	0.16	3.579	0.149	20341.323	10288.823
Week 21	0.451	0.052	28.751	14.599	100.749	56.272	0.477	0.308	3.669	0.216	24168.791	26775.167
	0.431	0.05	25.436	7.145	90.71	30.192	0.428	0.166	3.516	0.129	21275.255	8067.3
Week 22	0.434	0.058	27.902	8.367	100.412	33.925	0.469	0.186	3.574	0.188	20392.81	10815.921
	0.441	0.049	24.745	6.915	87.229	26.18	0.424	0.158	3.541	0.118	19548.73	10279.678
Week 23	0.466	0.071	24.189	13.363	82.847	42.529	0.399	0.254	3.641	0.218	14630.54	9271.917
	0.452	0.05	24.961	6.567	87.299	28.566	0.43	0.168	3.565	0.118	18967.674	8166.131
Week 24	0.429	0.047	22.683	8.933	80.953	35.813	0.385	0.222	3.566	0.205	17707.272	12064.405
	0.448	0.064	24.349	10.124	86.571	43.179	0.428	0.256	3.561	0.158	20683.086	12424.134

Table S3. Descriptive statistics for the kinematics (median).

Variable	Duration		Range		Angular Velocity		Angular Acceleration		Entropy		Jerk	
	Mean	SD	Mean	SD	Mean	SD	Mean	SD	Mean	SD	Mean	SD
Week 5	0.39	0.064	20.03	5.812	67.755	23.132	0.281	0.078	3.557	0.18	16405.233	11741.382
	0.426	0.042	15.891	5.193	52.262	18.907	0.22	0.079	3.673	0.103	10643.797	3472.559
Week 6	0.394	0.066	15.526	4.825	52.586	20.318	0.224	0.094	3.592	0.183	13485.595	9195.793
	0.413	0.067	15.133	3.951	50.886	16.2	0.215	0.067	3.651	0.17	11134.221	5230.034
Week 7	0.421	0.055	16.12	4.274	53.81	17.768	0.212	0.06	3.652	0.166	10894.597	6039.264
	0.39	0.054	16.834	10.123	60.152	38.685	0.26	0.184	3.579	0.164	27075.444	65641.393
Week 8	0.391	0.04	17.01	6.332	57.907	23.728	0.233	0.088	3.573	0.139	15670.822	12819.721
	0.379	0.062	16.741	5.67	59.681	23.052	0.238	0.089	3.552	0.179	13086.102	6397.188
Week 9	0.383	0.07	15.352	5.423	54.496	22.687	0.212	0.083	3.579	0.299	12710.102	5503.57
	0.398	0.056	15.178	4.999	52.22	18.371	0.226	0.084	3.614	0.141	13307.62	6082.969
Week 10	0.367	0.049	14.908	4.902	53.652	17.922	0.226	0.071	3.672	0.375	12904.118	6740.859
	0.354	0.047	16.949	3.056	63.132	13.706	0.264	0.074	3.467	0.151	18964.06	7489.838
Week 11	0.364	0.034	14.074	2.577	49.928	9.536	0.215	0.042	3.626	0.302	12926.303	4731.648
	0.361	0.045	13.308	4.027	47.941	16.193	0.202	0.074	3.522	0.125	11340.756	4262.35
Week 12	0.341	0.032	14.348	3.972	52.054	16.105	0.207	0.07	3.497	0.259	13296.474	6549.68
	0.35	0.038	15.19	3.249	55.653	13.12	0.225	0.046	3.486	0.131	14458.523	6141.711
Week 13	0.358	0.049	11.887	2.827	42.7	11.648	0.181	0.049	3.587	0.313	12738.619	5974.506
	0.352	0.036	14.283	3.149	51.004	12.346	0.211	0.05	3.497	0.143	13736.866	4165.884
Week 14	0.357	0.04	13.308	6.052	47.366	21.568	0.19	0.082	3.521	0.249	13427.206	5996.516
	0.364	0.052	13.7	3.452	48.644	13.362	0.202	0.057	3.519	0.235	13196.813	6039.377
Week 15	0.363	0.043	13.552	4.399	47.163	16.567	0.193	0.074	3.544	0.197	13049.912	5719.671
	0.359	0.038	12.693	4.165	45.319	16.754	0.184	0.07	3.499	0.134	12638.107	6522.981
Week 16	0.357	0.054	14.299	5.802	51.414	23.093	0.199	0.087	3.581	0.27	14574.409	7596.188
	0.357	0.039	13.287	4.851	47.155	19.373	0.197	0.08	3.501	0.141	12971.414	6717.908
Week 17	0.362	0.03	12.262	4.313	43.233	17.145	0.171	0.065	3.509	0.105	11844.213	6565.857
	0.359	0.024	13.403	3.599	47.17	13.43	0.2	0.056	3.511	0.079	12612.672	5340.102
Week 18	0.371	0.052	13.74	4.605	48.057	18.444	0.189	0.073	3.634	0.357	13309.385	5541.088
	0.382	0.069	14.111	13.44	47.973	46.476	0.199	0.217	3.563	0.207	20966.861	49187.341
Week 19	0.373	0.037	11.367	2.753	39.854	11.912	0.165	0.053	3.721	0.336	11200.14	4694.101
	0.381	0.04	11.946	3.326	40.543	11.847	0.166	0.048	3.563	0.118	10791.09	4341.731
Week 20	0.401	0.064	11.316	3.232	37.722	11.687	0.15	0.042	3.62	0.171	9664.832	2997.777
	0.389	0.062	13.435	5.203	46.232	19.371	0.186	0.068	3.555	0.191	12148.666	6355.352
Week 21	0.377	0.055	14.441	8.307	52.23	37.064	0.213	0.149	3.666	0.241	15132.765	15063.766
	0.363	0.049	13.968	3.969	49.512	15.778	0.198	0.06	3.509	0.15	13282.222	4881.348
Week 22	0.359	0.054	12.496	2.878	43.624	12.536	0.179	0.055	3.566	0.205	11427.557	5296.799
	0.37	0.053	12.641	3.029	43.668	11.413	0.176	0.048	3.524	0.154	11924.617	5931.11
Week 23	0.401	0.061	11.033	3.463	37.365	13.93	0.155	0.07	3.644	0.242	8958.622	5714.526
	0.379	0.043	12.756	3.165	44.431	12.894	0.182	0.056	3.564	0.14	11592.82	4945.409
Week 24	0.357	0.04	11.018	4.085	38.273	15.902	0.158	0.073	3.555	0.245	11857.561	9483.204
	0.38	0.059	12.817	5.022	45.053	23.449	0.189	0.112	3.551	0.191	12670.142	7663.745

Table S4. Descriptive statistics for the kinematics (max).

Variable	Duration		Range		Angular Velocity		Angular Acceleration		Entropy		Jerk	
	Mean	SD	Mean	SD	Mean	SD	Mean	SD	Mean	SD	Mean	SD
Week 5	1.892	0.09	240.413	43.248	1026.372	127.251	7.751	1.988	5.458	0.153	383726.887	777391.366
	1.917	0.08	241.925	39.168	993.884	216.288	7.524	2.232	5.451	0.114	162045.998	131840.989
Week 6	1.885	0.12	267.24	43.027	1206.458	154.327	9.142	1.723	5.38	0.109	275718.618	389497.641
	1.829	0.148	225.123	34.187	954.372	199.69	7.26	3.055	5.417	0.137	123445.257	85002.854
Week 7	1.911	0.124	240.503	41.258	1048.206	182.539	7.61	1.977	5.463	0.18	125534.303	67630.876
	1.832	0.167	237.723	35.771	1050.21	150.341	8.481	1.532	5.377	0.197	196880.972	210705.203
Week 8	1.832	0.128	241.695	51.307	1030.245	209.095	7.944	2.117	5.326	0.175	209853.333	243304.996
	1.82	0.18	230.824	40.483	1007.94	190.498	7.31	2.215	5.375	0.139	198469.748	153801.386
Week 9	1.842	0.158	239.749	61.531	1087.576	375.265	7.338	2.446	5.427	0.244	192174.792	214634.236
	1.811	0.115	214.901	23.16	972.461	122.616	7.614	1.924	5.345	0.125	117446.522	45764.373
Week 10	1.739	0.198	252.24	36.507	1131.383	194.456	7.901	1.527	5.406	0.238	156985.85	119733.265
	1.763	0.218	233.2	31.163	1028.849	144.627	8.312	1.908	5.295	0.193	279125.741	328771.613
Week 11	1.801	0.161	235.055	44.016	1016.671	240.25	7.85	2.412	5.394	0.213	158749.693	118370.289
	1.815	0.165	219.765	60.878	991.555	303.473	7.283	2.59	5.388	0.181	188014.82	187673.478
Week 12	1.742	0.143	224.994	45.028	1010.525	287.932	7.298	2.825	5.377	0.191	161970.296	86994.309
	1.801	0.174	217.729	47.791	976.926	203.843	7.4	2.418	5.408	0.123	181487.538	157731.529
Week 13	1.765	0.187	229.813	48.814	1037.811	320.122	7.159	2.852	5.343	0.153	173604.802	153495.42
	1.796	0.145	231.833	43.723	986.811	239.312	7.681	2.511	5.439	0.167	208499.817	191614.849
Week 14	1.782	0.144	222.736	49.475	1007.684	304.158	7.029	2.9	5.359	0.159	145850.453	83495.176
	1.751	0.178	219.057	39.353	1000.867	247.612	7.921	2.075	5.373	0.175	128169.543	42110.53
Week 15	1.818	0.132	226.347	42.248	996.327	244.348	7.222	2.709	5.408	0.219	159966.192	94225.991
	1.718	0.153	218.362	56.869	938.906	258.969	7.095	2.601	5.366	0.16	244144.668	263991.233
Week 16	1.773	0.122	229.107	53.042	1006.715	314.311	6.9	3.19	5.456	0.181	138959.301	54431.521
	1.773	0.189	220.297	51.371	958.328	266.828	7.218	2.611	5.353	0.14	177155.593	140387.172
Week 17	1.778	0.18	236.131	82.185	1037.159	434.756	6.793	3.436	5.394	0.173	187784.015	135091.871
	1.786	0.174	226.138	42.776	996.168	267.748	7.344	3.229	5.422	0.151	197530.671	230144.779
Week 18	1.8	0.193	251.212	60.499	1069.208	331.101	7.632	2.892	5.495	0.197	222644.295	245412.788
	1.81	0.215	221.208	64.261	960.986	288.909	6.933	2.804	5.431	0.173	252943.273	315334.988
Week 19	1.722	0.17	206.086	53.068	891.032	311.767	6.373	3.226	5.351	0.234	150685.656	82186.386
	1.852	0.103	219.955	46.208	920.34	222.362	6.841	2.388	5.451	0.166	129664.924	47678.389
Week 20	1.792	0.15	222.144	58.411	920.143	265.336	5.645	1.955	5.379	0.131	173081.37	176027.077
	1.817	0.139	211.984	71.068	906.158	343.33	6.295	2.615	5.392	0.143	175508.272	150680.908
Week 21	1.876	0.106	244.059	69.237	1047.334	367.823	7.386	4.055	5.437	0.151	174341.921	157705.495
	1.821	0.157	225.767	55.883	962.096	315.678	6.773	3.175	5.449	0.143	191887.111	134224.27
Week 22	1.759	0.17	243.537	57.974	1030.123	298.983	7.031	2.738	5.412	0.188	241624.077	225382.112
	1.759	0.198	229.809	63.385	1020.408	347.548	7.167	3.236	5.37	0.143	179383.387	119299.608
Week 23	1.707	0.162	186.796	58.169	801.185	302.428	5.596	2.743	5.337	0.204	116072.666	71601.754
	1.802	0.158	238.675	31.5	1003.07	160.35	6.749	2.359	5.375	0.178	180888.009	131975.732
Week 24	1.716	0.152	192.906	66.67	855.399	368.698	5.689	3.512	5.358	0.208	135018.11	79320.746
	1.795	0.193	214.318	66.251	871.396	309.573	6.245	2.932	5.389	0.177	184003.275	133505.263

Table S5. Descriptive statistics for the kinematics (variability coefficient).

Variable	Duration		Range		Angular Velocity		Angular Acceleration		Entropy		Jerk	
	Mean	SD	Mean	SD	Mean	SD	Mean	SD	Mean	SD	Mean	SD
Week 5	0.595	0.025	1.178	0.175	1.289	0.216	1.513	0.191	0.171	0.006	1.133	0.505
	0.592	0.024	1.263	0.213	1.399	0.273	1.665	0.311	0.168	0.007	1.101	0.343
Week 6	0.604	0.038	1.309	0.156	1.456	0.204	1.743	0.238	0.174	0.012	1.155	0.254
	0.576	0.029	1.282	0.216	1.421	0.292	1.651	0.3	0.166	0.007	1.016	0.175
Week 7	0.574	0.023	1.247	0.202	1.378	0.245	1.661	0.28	0.164	0.008	1.043	0.111
	0.571	0.024	1.302	0.199	1.433	0.272	1.713	0.299	0.165	0.005	1.041	0.264
Week 8	0.578	0.035	1.271	0.178	1.381	0.235	1.692	0.284	0.167	0.01	1.051	0.309
	0.561	0.036	1.202	0.194	1.296	0.235	1.611	0.253	0.163	0.006	1.127	0.358
Week 9	0.562	0.033	1.281	0.276	1.379	0.334	1.645	0.398	0.161	0.016	1.054	0.283
	0.56	0.024	1.215	0.249	1.355	0.33	1.637	0.343	0.162	0.005	0.932	0.097
Week 10	0.557	0.046	1.352	0.246	1.469	0.307	1.692	0.335	0.156	0.027	1.028	0.359
	0.57	0.04	1.156	0.123	1.234	0.159	1.568	0.258	0.167	0.008	1.035	0.394
Week 11	0.561	0.031	1.303	0.191	1.396	0.215	1.635	0.25	0.157	0.02	1.008	0.213
	0.566	0.03	1.233	0.217	1.326	0.249	1.66	0.284	0.167	0.005	1.143	0.476
Week 12	0.561	0.038	1.246	0.216	1.333	0.271	1.616	0.341	0.162	0.016	0.981	0.182
	0.576	0.024	1.186	0.154	1.265	0.2	1.542	0.233	0.168	0.006	1.02	0.184
Week 13	0.569	0.029	1.351	0.159	1.465	0.216	1.7	0.292	0.162	0.02	1.093	0.324
	0.578	0.026	1.205	0.204	1.262	0.255	1.554	0.305	0.171	0.006	1.09	0.348
Week 14	0.57	0.031	1.304	0.321	1.377	0.347	1.676	0.382	0.167	0.017	0.997	0.178
	0.584	0.029	1.298	0.246	1.41	0.299	1.764	0.336	0.171	0.015	0.963	0.106
Week 15	0.592	0.04	1.313	0.213	1.407	0.254	1.678	0.315	0.171	0.015	1.04	0.359
	0.576	0.035	1.327	0.269	1.441	0.337	1.789	0.378	0.172	0.007	1.28	0.713
Week 16	0.578	0.026	1.269	0.29	1.37	0.378	1.629	0.412	0.166	0.016	0.952	0.129
	0.592	0.042	1.316	0.267	1.446	0.359	1.714	0.401	0.173	0.009	1.092	0.392
Week 17	0.597	0.035	1.319	0.256	1.405	0.309	1.699	0.389	0.177	0.009	1.146	0.375
	0.593	0.026	1.307	0.203	1.409	0.265	1.689	0.304	0.175	0.006	1.07	0.364
Week 18	0.595	0.035	1.35	0.206	1.462	0.293	1.769	0.338	0.167	0.022	1.184	0.549
	0.59	0.041	1.335	0.276	1.47	0.348	1.777	0.45	0.173	0.008	1.142	0.344
Week 19	0.585	0.031	1.261	0.179	1.361	0.212	1.634	0.228	0.163	0.023	1.067	0.114
	0.601	0.027	1.352	0.315	1.478	0.387	1.809	0.455	0.176	0.007	1.041	0.141
Week 20	0.571	0.028	1.343	0.289	1.491	0.392	1.737	0.397	0.169	0.006	1.116	0.284
	0.583	0.039	1.214	0.302	1.313	0.364	1.625	0.426	0.173	0.008	1.101	0.352
Week 21	0.596	0.033	1.388	0.273	1.497	0.33	1.693	0.308	0.168	0.02	1.022	0.134
	0.594	0.029	1.196	0.196	1.276	0.239	1.571	0.303	0.175	0.007	1.097	0.287
Week 22	0.593	0.026	1.4	0.232	1.493	0.283	1.705	0.316	0.173	0.014	1.208	0.397
	0.601	0.038	1.342	0.289	1.47	0.385	1.738	0.394	0.175	0.005	1.142	0.265
Week 23	0.582	0.04	1.226	0.273	1.327	0.379	1.612	0.484	0.173	0.018	1.104	0.257
	0.599	0.031	1.344	0.161	1.458	0.204	1.748	0.188	0.176	0.007	1.131	0.378
Week 24	0.577	0.032	1.362	0.387	1.474	0.426	1.681	0.453	0.169	0.013	1.032	0.236
	0.581	0.04	1.291	0.277	1.381	0.33	1.656	0.399	0.172	0.007	1.109	0.211

S 4

GLMM results

Table S6. The results of GLMMs for kinematic measures (mean).

Variable	Effect	Coefficient	SE	Z Value	sig	Effect Size	CI (95%)	
							Lower	Higher
Duration	Group	0.0084	0.0376	0.2224	0.8916	1.0084	-0.0654	0.0821
	Age (Week): Quadratic	0.0015	4.00E-04	3.9893	< .001	1.0015	7.00E-04	0.0022
	Age (Week): Linear	-0.0022	0.002	-1.138	0.464	0.9978	-0.0061	0.0016
	Gestational Age	-0.0025	0.011	-0.2324	0.8916	0.9975	-0.024	0.0189
	Group × Age (Week): Quadratic	1.00E-04	5.00E-04	0.1322	0.9211	1.0001	-9.00E-04	0.001
	Group × Age (Week): Linear	-2.00E-04	0.0025	-0.0665	0.9561	0.9998	-0.0051	0.0048
Range	Group	-0.005	0.05	-0.1009	0.9375	0.995	-0.1031	0.093
	Age (Week): Quadratic	0.0021	6.00E-04	3.7834	< .001	1.0021	0.001	0.0032
	Age (Week): Linear	-0.0171	0.0029	-5.858	< .001	0.983	-0.0228	-0.0114
	Gestational Age	0.0126	0.0143	0.8791	0.5969	1.0126	-0.0154	0.0406
	Group × Age (Week): Quadratic	-0.001	7.00E-04	-1.3699	0.3658	0.999	-0.0024	4.00E-04
	Group × Age (Week): Linear	0.0053	0.0037	1.4231	0.3456	1.0053	-0.002	0.0125
Angular Velocity	Group	0.0197	0.0526	0.3741	0.8548	1.0199	-0.0835	0.1229
	Age (Week): Quadratic	0.0025	6.00E-04	3.9739	< .001	1.0025	0.0013	0.0037
	Age (Week): Linear	-0.0172	0.0033	-5.2497	< .001	0.9829	-0.0237	-0.0108
	Gestational Age	0.0099	0.0147	0.674	0.6912	1.01	-0.0189	0.0388
	Group × Age (Week): Quadratic	-0.0019	8.00E-04	-2.3232	0.073	0.9981	-0.0034	-3.00E-04
	Group × Age (Week): Linear	0.0035	0.0042	0.8332	0.6131	1.0035	-0.0047	0.0118
Angular Acceleration	Group	0.0805	0.0626	1.2853	0.4091	1.0838	-0.0423	0.2033
	Age (Week): Quadratic	0.0032	7.00E-04	4.3841	< .001	1.0032	0.0018	0.0047
	Age (Week): Linear	-0.0226	0.0039	-5.8425	< .001	0.9777	-0.0301	-0.015
	Gestational Age	0.0108	0.0176	0.6133	0.7359	1.0108	-0.0237	0.0453
	Group × Age (Week): Quadratic	-0.0027	9.00E-04	-2.8329	0.0186	0.9973	-0.0045	-8.00E-04
	Group × Age (Week): Linear	0.0052	0.005	1.0567	0.4923	1.0053	-0.0045	0.015
Entropy	Group	-0.0105	0.0099	-1.0618	0.5628	0.9896	-0.0298	0.0089
	Age (Week): Quadratic	1.00E-04	1.00E-04	1.1416	0.5326	1.0001	-1.00E-04	2.00E-04
	Age (Week): Linear	1.00E-04	4.00E-04	0.2196	0.9728	1.0001	-7.00E-04	8.00E-04
	Gestational Age	1.00E-04	0.003	0.0306	0.9787	1.0001	-0.0057	0.0059
	Group × Age (Week): Quadratic	1.00E-04	1.00E-04	1.472	0.4368	1.0001	0	3.00E-04
	Group × Age (Week): Linear	-7.00E-04	5.00E-04	-1.474	0.4368	0.9993	-0.0017	2.00E-04
Jerk	Group	0.0273	0.0832	0.3284	0.8568	1.0277	-0.1358	0.1905
	Age (Week): Quadratic	-7.00E-04	9.00E-04	-0.8623	0.5999	0.9993	-0.0024	9.00E-04
	Age (Week): Linear	-0.008	0.0047	-1.7268	0.2377	0.992	-0.0172	0.0011
	Gestational Age	0.021	0.0239	0.8763	0.5969	1.0212	-0.026	0.0679
	Group × Age (Week): Quadratic	-5.00E-04	0.0011	-0.4581	0.8108	0.9995	-0.0027	0.0017
	Group × Age (Week): Linear	0.0071	0.0059	1.1968	0.4499	1.0071	-0.0045	0.0187

Table S7. The results of GLMMs for kinematic measures (median).

Variable	Effect	Coefficient	SE	Z Value	sig	Effect Size	CI (95%)	
							Lower	Higher
Duration	Group	9.00E-04	0.0351	0.0243	0.9806	1.0009	-0.068	0.0697
	Age (Week): Quadratic	0.0013	4.00E-04	3.438	0.0028	1.0013	6.00E-04	0.002
	Age (Week): Linear	-0.0032	0.002	-1.5967	0.2758	0.9968	-0.0071	7.00E-04
	Gestational Age	-0.0026	0.0101	-0.2559	0.8916	0.9974	-0.0223	0.0171
	Group × Age (Week): Quadratic	4.00E-04	5.00E-04	0.8261	0.6131	1.0004	-6.00E-04	0.0014
	Group × Age (Week): Linear	-5.00E-04	0.0026	-0.1782	0.9106	0.9995	-0.0055	0.0046
Range	Group	0.0106	0.0501	0.212	0.8916	1.0107	-0.0876	0.1088
	Age (Week): Quadratic	0.0012	5.00E-04	2.3352	0.073	1.0012	2.00E-04	0.0023
	Age (Week): Linear	-0.0184	0.0028	-6.5234	< .001	0.9818	-0.0239	-0.0128
	Gestational Age	0.0108	0.0144	0.7469	0.6547	1.0108	-0.0175	0.039
	Group × Age (Week): Quadratic	-6.00E-04	7.00E-04	-0.913	0.5835	0.9994	-0.002	7.00E-04
	Group × Age (Week): Linear	0.0075	0.0036	2.0852	0.1183	1.0075	5.00E-04	0.0146
Angular Velocity	Group	0.0184	0.0542	0.3402	0.8568	1.0186	-0.0878	0.1246
	Age (Week): Quadratic	8.00E-04	6.00E-04	1.2915	0.4091	1.0008	-4.00E-04	0.0019
	Age (Week): Linear	-0.0192	0.0032	-6.0574	< .001	0.981	-0.0253	-0.013
	Gestational Age	0.0104	0.0154	0.6759	0.6912	1.0105	-0.0198	0.0406
	Group × Age (Week): Quadratic	-9.00E-04	8.00E-04	-1.1749	0.4583	0.9991	-0.0024	6.00E-04
	Group × Age (Week): Linear	0.0069	0.0041	1.691	0.2446	1.0069	-0.0011	0.0148
Angular Acceleration	Group	0.0319	0.0651	0.4909	0.7983	1.0325	-0.0956	0.1595
	Age (Week): Quadratic	0.0012	8.00E-04	1.6078	0.2758	1.0012	-3.00E-04	0.0027
	Age (Week): Linear	-0.0245	0.004	-6.088	< .001	0.9758	-0.0324	-0.0166
	Gestational Age	0.0214	0.0184	1.1636	0.4586	1.0216	-0.0146	0.0574
	Group × Age (Week): Quadratic	-0.0014	0.001	-1.4856	0.3136	0.9986	-0.0033	5.00E-04
	Group × Age (Week): Linear	0.0065	0.0051	1.2644	0.4161	1.0065	-0.0036	0.0165
Entropy	Group	-0.0115	0.0112	-1.0285	0.5628	0.9885	-0.0335	0.0104
	Age (Week): Quadratic	1.00E-04	1.00E-04	1.4591	0.4368	1.0001	0	3.00E-04
	Age (Week): Linear	2.00E-04	5.00E-04	0.3956	0.9483	1.0002	-7.00E-04	0.0011
	Gestational Age	3.00E-04	0.0034	0.092	0.9728	1.0003	-0.0063	0.0069
	Group × Age (Week): Quadratic	1.00E-04	1.00E-04	1.3184	0.4714	1.0001	-1.00E-04	4.00E-04
	Group × Age (Week): Linear	-0.001	6.00E-04	-1.6399	0.4368	0.999	-0.0021	2.00E-04
Jerk	Group	-0.0288	0.0828	-0.3484	0.8568	0.9716	-0.1911	0.1334
	Age (Week): Quadratic	-0.0018	8.00E-04	-2.1496	0.107	0.9982	-0.0034	-2.00E-04
	Age (Week): Linear	-0.0083	0.0045	-1.8569	0.19	0.9917	-0.0171	5.00E-04
	Gestational Age	0.0233	0.024	0.9684	0.5461	1.0235	-0.0238	0.0704
	Group × Age (Week): Quadratic	8.00E-04	0.0011	0.7798	0.6441	1.0008	-0.0013	0.0029
	Group × Age (Week): Linear	0.0043	0.0057	0.7586	0.6535	1.0043	-0.0069	0.0155

Table S8. The results of GLMM for kinematic measures (maximum).

Variable	Effect	Coefficient	SE	Z Value	sig	Effect Size	CI (95%)	
							Lower	Higher
Duration	Group	0.0046	0.0136	0.336	0.8568	1.0046	-0.0222	0.0313
	Age (Week): Quadratic	2.00E-04	2.00E-04	1.088	0.4761	1.0002	-2.00E-04	6.00E-04
	Age (Week): Linear	-0.0031	0.001	-3.072	0.0089	0.9969	-0.005	-0.0011
	Gestational Age	-0.0041	0.0037	-1.1322	0.464	0.9959	-0.0113	0.003
	Group × Age (Week): Quadratic	2.00E-04	2.00E-04	0.6834	0.6912	1.0002	-3.00E-04	6.00E-04
	Group × Age (Week): Linear	0.0022	0.0013	1.7668	0.2254	1.0022	-2.00E-04	0.0047
Range	Group	-0.0831	0.0381	-2.1806	0.1023	0.9202	-0.1579	-0.0084
	Age (Week): Quadratic	-1.00E-04	5.00E-04	-0.1375	0.9211	0.9999	-0.0011	9.00E-04
	Age (Week): Linear	-0.0074	0.0027	-2.7412	0.0238	0.9926	-0.0127	-0.0021
	Gestational Age	0.0155	0.0103	1.5074	0.3073	1.0156	-0.0047	0.0357
	Group × Age (Week): Quadratic	7.00E-04	7.00E-04	1.1041	0.4717	1.0007	-6.00E-04	0.002
	Group × Age (Week): Linear	0.0059	0.0034	1.7168	0.2377	1.0059	-8.00E-04	0.0126
Angular Velocity	Group	-0.0476	0.0423	-1.1247	0.464	0.9535	-0.1306	0.0354
	Age (Week): Quadratic	1.00E-04	6.00E-04	0.1622	0.9147	1.0001	-0.0011	0.0013
	Age (Week): Linear	-0.0108	0.0033	-3.2797	0.0047	0.9893	-0.0172	-0.0043
	Gestational Age	0.0152	0.011	1.385	0.3633	1.0153	-0.0063	0.0367
	Group × Age (Week): Quadratic	-2.00E-04	8.00E-04	-0.2991	0.8729	0.9998	-0.0018	0.0013
	Group × Age (Week): Linear	0.0067	0.0041	1.6216	0.2753	1.0067	-0.0014	0.0148
Angular Acceleration	Group	0.0285	0.055	0.5184	0.7983	1.0289	-0.0793	0.1364
	Age (Week): Quadratic	3.00E-04	9.00E-04	0.3948	0.8461	1.0003	-0.0014	0.002
	Age (Week): Linear	-0.0185	0.0046	-4.0506	< .001	0.9817	-0.0274	-0.0095
	Gestational Age	0.0286	0.0137	2.0839	0.1183	1.0291	0.0017	0.0556
	Group × Age (Week): Quadratic	-0.0011	0.0011	-1.0176	0.5148	0.9989	-0.0033	0.001
	Group × Age (Week): Linear	0.0087	0.0058	1.5155	0.3073	1.0088	-0.0026	0.0201
Entropy	Group	7.00E-04	0.0047	0.1595	0.9728	1.0007	-0.0084	0.0099
	Age (Week): Quadratic	0	1.00E-04	0.161	0.9728	1	-1.00E-04	1.00E-04
	Age (Week): Linear	-2.00E-04	3.00E-04	-0.507	0.9062	0.9998	-8.00E-04	5.00E-04
	Gestational Age	-2.00E-04	0.0013	-0.1437	0.9728	0.9998	-0.0027	0.0023
	Group × Age (Week): Quadratic	0	1.00E-04	0.2987	0.9641	1	-1.00E-04	2.00E-04
	Group × Age (Week): Linear	3.00E-04	4.00E-04	0.837	0.7152	1.0003	-5.00E-04	0.0012
Jerk	Group	-0.0517	0.1038	-0.4985	0.7983	0.9496	-0.2551	0.1517
	Age (Week): Quadratic	-6.00E-04	0.0013	-0.44	0.8152	0.9994	-0.0031	0.002
	Age (Week): Linear	0.0015	0.0067	0.2198	0.8916	1.0015	-0.0117	0.0146
	Gestational Age	0.007	0.0288	0.2432	0.8916	1.007	-0.0495	0.0635
	Group × Age (Week): Quadratic	9.00E-04	0.0016	0.5744	0.7615	1.0009	-0.0022	0.0041
	Group × Age (Week): Linear	0.0043	0.0084	0.5092	0.7983	1.0043	-0.0122	0.0208

Table S9. The results of GLMMs for kinematic measures (variability).

Variable	Effect	Coefficient	SE	Z Value	sig	Effect Size	CI (95%)	
							Lower	Higher
Duration	Group	0.0314	0.02	1.5727	0.4368	1.0319	-0.0077	0.0706
	Age (Week): Quadratic	8.00E-04	3.00E-04	3.1333	0.0136	1.0008	3.00E-04	0.0013
	Age (Week): Linear	0.0032	0.0014	2.3086	0.1321	1.0032	5.00E-04	0.0059
	Gestational Age	4.00E-04	0.0054	0.0728	0.9728	1.0004	-0.0102	0.011
	Group × Age (Week): Quadratic	-5.00E-04	3.00E-04	-1.5802	0.4368	0.9995	-0.0012	1.00E-04
	Group × Age (Week): Linear	0.0031	0.0018	1.7422	0.4277	1.0031	-4.00E-04	0.0066
Range	Group	-0.0476	0.0237	-2.0076	0.2559	0.9535	-0.0941	-0.0011
	Age (Week): Quadratic	-4.00E-04	4.00E-04	-1.0983	0.553	0.9996	-0.0011	3.00E-04
	Age (Week): Linear	0.0029	0.0019	1.5172	0.4368	1.0029	-8.00E-04	0.0066
	Gestational Age	0.002	0.006	0.3334	0.9635	1.002	-0.0098	0.0138
	Group × Age (Week): Quadratic	6.00E-04	5.00E-04	1.1999	0.5	1.0006	-4.00E-04	0.0015
	Group × Age (Week): Linear	-6.00E-04	0.0024	-0.2683	0.9728	0.9994	-0.0054	0.0041
Angular Velocity	Group	-0.0358	0.028	-1.2758	0.4714	0.9649	-0.0907	0.0192
	Age (Week): Quadratic	-3.00E-04	4.00E-04	-0.708	0.7737	0.9997	-0.0011	5.00E-04
	Age (Week): Linear	9.00E-04	0.0022	0.422	0.9483	1.0009	-0.0034	0.0052
	Gestational Age	-7.00E-04	0.0072	-0.1028	0.9728	0.9993	-0.0149	0.0134
	Group × Age (Week): Quadratic	6.00E-04	5.00E-04	1.0412	0.5628	1.0006	-5.00E-04	0.0016
	Group × Age (Week): Linear	6.00E-04	0.0028	0.2057	0.9728	1.0006	-0.0049	0.006
Angular Acceleration	Group	-0.0157	0.0262	-0.5989	0.844	0.9844	-0.0672	0.0357
	Age (Week): Quadratic	-6.00E-04	4.00E-04	-1.4874	0.4368	0.9994	-0.0014	2.00E-04
	Age (Week): Linear	-2.00E-04	0.0021	-0.106	0.9728	0.9998	-0.0043	0.0038
	Gestational Age	0.0048	0.0067	0.7116	0.7737	1.0048	-0.0084	0.0179
	Group × Age (Week): Quadratic	4.00E-04	5.00E-04	0.7775	0.7439	1.0004	-6.00E-04	0.0014
	Group × Age (Week): Linear	0.0011	0.0026	0.4043	0.9483	1.0011	-0.0041	0.0063
Entropy	Group	0.0581	0.0206	2.8215	0.0335	1.0598	0.0177	0.0985
	Age (Week): Quadratic	6.00E-04	1.00E-04	3.9576	< .001	1.0006	3.00E-04	8.00E-04
	Age (Week): Linear	0.0033	8.00E-04	4.3433	< .001	1.0033	0.0018	0.0048
	Gestational Age	-0.0031	0.0062	-0.498	0.9062	0.9969	-0.0153	0.0091
	Group × Age (Week): Quadratic	-7.00E-04	2.00E-04	-3.6515	0.0023	0.9993	-0.001	-3.00E-04
	Group × Age (Week): Linear	0.0012	0.001	1.2464	0.4784	1.0012	-7.00E-04	0.0031
Jerk	Group	0.0194	0.0311	0.6216	0.8414	1.0195	-0.0417	0.0804
	Age (Week): Quadratic	6.00E-04	4.00E-04	1.4306	0.4368	1.0006	-2.00E-04	0.0014
	Age (Week): Linear	0.0031	0.0022	1.4459	0.4368	1.0032	-0.0011	0.0074
	Gestational Age	-2.00E-04	0.0081	-0.0267	0.9787	0.9998	-0.0162	0.0157

	**Group × Age (Week): Quadratic**	-2.00E-04	6.00E-04	-0.3614	0.9621	0.9998	-0.0013	9.00E-04
	**Group × Age (Week): Linear**	9.00E-04	0.0029	0.3195	0.9635	1.0009	-0.0048	0.0066

S 5

Marginal contrasts

Age_Week	Estimated_Diff	p_raw	p_adj	Statistic	Kinematic	xmin	xmax
5	0.009129	0.751051	0.816744	Mean	Duration	4.5	5.5
6	0.008304	0.745783	0.816744	Mean	Duration	5.5	6.5
7	0.007568	0.745265	0.816744	Mean	Duration	6.5	7.5
8	0.006919	0.750527	0.816744	Mean	Duration	7.5	8.5
9	0.006358	0.761001	0.816744	Mean	Duration	8.5	9.5
10	0.005884	0.774598	0.816744	Mean	Duration	9.5	10.5
11	0.005498	0.788676	0.816744	Mean	Duration	10.5	11.5
12	0.0052	0.801025	0.816744	Mean	Duration	11.5	12.5
13	0.00499	0.810233	0.816744	Mean	Duration	12.5	13.5
14	0.004867	0.81557	0.816744	Mean	Duration	13.5	14.5
15	0.004832	0.816744	0.816744	Mean	Duration	14.5	15.5
16	0.004885	0.813737	0.816744	Mean	Duration	15.5	16.5
17	0.005025	0.806792	0.816744	Mean	Duration	16.5	17.5
18	0.005253	0.796548	0.816744	Mean	Duration	17.5	18.5
19	0.005569	0.784279	0.816744	Mean	Duration	18.5	19.5
20	0.005973	0.77205	0.816744	Mean	Duration	19.5	20.5
21	0.006464	0.762429	0.816744	Mean	Duration	20.5	21.5
22	0.007043	0.757571	0.816744	Mean	Duration	21.5	22.5
23	0.007709	0.758187	0.816744	Mean	Duration	22.5	23.5
24	0.008463	0.763373	0.816744	Mean	Duration	23.5	24.5
5	0.026569	0.399292	0.952458	Median	Duration	4.5	5.5
6	0.021744	0.432375	0.952458	Median	Duration	5.5	6.5
7	0.01743	0.483098	0.952458	Median	Duration	6.5	7.5
8	0.013627	0.552866	0.952458	Median	Duration	7.5	8.5
9	0.010334	0.636989	0.952458	Median	Duration	8.5	9.5
10	0.007552	0.724985	0.952458	Median	Duration	9.5	10.5
11	0.005281	0.805417	0.952458	Median	Duration	10.5	11.5
12	0.003521	0.870519	0.952458	Median	Duration	11.5	12.5
13	0.002272	0.916983	0.952458	Median	Duration	12.5	13.5
14	0.001533	0.944213	0.952458	Median	Duration	13.5	14.5
15	0.001305	0.952458	0.952458	Median	Duration	14.5	15.5
16	0.001588	0.941768	0.952458	Median	Duration	15.5	16.5
17	0.002381	0.911804	0.952458	Median	Duration	16.5	17.5
18	0.003686	0.8625	0.952458	Median	Duration	17.5	18.5
19	0.005501	0.795662	0.952458	Median	Duration	18.5	19.5
20	0.007827	0.717005	0.952458	Median	Duration	19.5	20.5
21	0.010664	0.636602	0.952458	Median	Duration	20.5	21.5
22	0.014011	0.565476	0.952458	Median	Duration	21.5	22.5
23	0.017869	0.510358	0.952458	Median	Duration	22.5	23.5
24	0.022238	0.471835	0.952458	Median	Duration	23.5	24.5
5	-0.00155	0.944449	0.984205	Max	Duration	4.5	5.5
6	-0.00236	0.900811	0.984205	Max	Duration	5.5	6.5
7	-0.00284	0.862446	0.984205	Max	Duration	6.5	7.5
8	-0.00298	0.838246	0.984205	Max	Duration	7.5	8.5
9	-0.0028	0.836974	0.984205	Max	Duration	8.5	9.5

10	-0.00228	0.862538	0.984205	Max	Duration	9.5	10.5
11	-0.00144	0.91301	0.984205	Max	Duration	10.5	11.5
12	-0.00026	0.984205	0.984205	Max	Duration	11.5	12.5
13	0.001241	0.926923	0.984205	Max	Duration	12.5	13.5
14	0.003077	0.821572	0.984205	Max	Duration	13.5	14.5
15	0.005244	0.70035	0.984205	Max	Duration	14.5	15.5
16	0.007741	0.565651	0.984205	Max	Duration	15.5	16.5
17	0.010568	0.424777	0.984205	Max	Duration	16.5	17.5
18	0.013726	0.292037	0.834392	Max	Duration	17.5	18.5
19	0.017215	0.185351	0.617837	Max	Duration	18.5	19.5
20	0.021033	0.115523	0.462091	Max	Duration	19.5	20.5
21	0.025183	0.079135	0.395677	Max	Duration	20.5	21.5
22	0.029662	0.065158	0.395677	Max	Duration	21.5	22.5
23	0.034473	0.064366	0.395677	Max	Duration	22.5	23.5
24	0.039613	0.071441	0.395677	Max	Duration	23.5	24.5
5	-0.01998	0.131208	0.344816	Variability	Duration	4.5	5.5
6	-0.01452	0.19961	0.362926	Variability	Duration	5.5	6.5
7	-0.00953	0.334554	0.477934	Variability	Duration	6.5	7.5
8	-0.005	0.574519	0.675904	Variability	Duration	7.5	8.5
9	-0.00092	0.9119	0.9119	Variability	Duration	8.5	9.5
10	0.002687	0.741735	0.780774	Variability	Duration	9.5	10.5
11	0.005838	0.474508	0.620556	Variability	Duration	10.5	11.5
12	0.008528	0.302405	0.469387	Variability	Duration	11.5	12.5
13	0.010757	0.199342	0.362926	Variability	Duration	12.5	13.5
14	0.012525	0.137926	0.344816	Variability	Duration	13.5	14.5
15	0.013833	0.100664	0.342912	Variability	Duration	14.5	15.5
16	0.014679	0.078126	0.342912	Variability	Duration	15.5	16.5
17	0.015065	0.06589	0.342912	Variability	Duration	16.5	17.5
18	0.01499	0.062994	0.342912	Variability	Duration	17.5	18.5
19	0.014454	0.072257	0.342912	Variability	Duration	18.5	19.5
20	0.013457	0.102874	0.342912	Variability	Duration	19.5	20.5
21	0.012	0.173499	0.362926	Variability	Duration	20.5	21.5
22	0.010082	0.305102	0.469387	Variability	Duration	21.5	22.5
23	0.007702	0.496445	0.620556	Variability	Duration	22.5	23.5
24	0.004862	0.714454	0.780774	Variability	Duration	23.5	24.5
5	-0.14792	0.041492	0.464279	Mean	Range	4.5	5.5
6	-0.12476	0.050119	0.464279	Mean	Range	5.5	6.5
7	-0.10354	0.069642	0.464279	Mean	Range	6.5	7.5
8	-0.08425	0.109444	0.547221	Mean	Range	7.5	8.5
9	-0.06691	0.181854	0.727417	Mean	Range	8.5	9.5
10	-0.05152	0.293835	0.979449	Mean	Range	9.5	10.5
11	-0.03806	0.437064	0.998589	Mean	Range	10.5	11.5
12	-0.02655	0.590469	0.998589	Mean	Range	11.5	12.5
13	-0.01698	0.733008	0.998589	Mean	Range	12.5	13.5
14	-0.00935	0.851798	0.998589	Mean	Range	13.5	14.5
15	-0.00366	0.941666	0.998589	Mean	Range	14.5	15.5

16	8.78E-05	0.998589	0.998589	Mean	Range	15.5	16.5
17	0.001892	0.969276	0.998589	Mean	Range	16.5	17.5
18	0.001754	0.971226	0.998589	Mean	Range	17.5	18.5
19	-0.00033	0.994639	0.998589	Mean	Range	18.5	19.5
20	-0.00435	0.929779	0.998589	Mean	Range	19.5	20.5
21	-0.01031	0.841536	0.998589	Mean	Range	20.5	21.5
22	-0.01822	0.743704	0.998589	Mean	Range	21.5	22.5
23	-0.02807	0.651099	0.998589	Mean	Range	22.5	23.5
24	-0.03985	0.57285	0.998589	Mean	Range	23.5	24.5
5	-0.1221	0.089819	0.883688	Median	Range	4.5	5.5
6	-0.10298	0.104338	0.883688	Median	Range	5.5	6.5
7	-0.08512	0.135458	0.883688	Median	Range	6.5	7.5
8	-0.06851	0.193814	0.883688	Median	Range	7.5	8.5
9	-0.05316	0.290329	0.883688	Median	Range	8.5	9.5
10	-0.03907	0.427463	0.883688	Median	Range	9.5	10.5
11	-0.02624	0.593141	0.883688	Median	Range	10.5	11.5
12	-0.01466	0.766746	0.891715	Median	Range	11.5	12.5
13	-0.00434	0.930545	0.930545	Median	Range	12.5	13.5
14	0.004717	0.92497	0.930545	Median	Range	13.5	14.5
15	0.012519	0.802543	0.891715	Median	Range	14.5	15.5
16	0.019063	0.70163	0.883688	Median	Range	15.5	16.5
17	0.024349	0.62112	0.883688	Median	Range	16.5	17.5
18	0.028377	0.560905	0.883688	Median	Range	17.5	18.5
19	0.031148	0.522527	0.883688	Median	Range	18.5	19.5
20	0.032661	0.508912	0.883688	Median	Range	19.5	20.5
21	0.032916	0.522871	0.883688	Median	Range	20.5	21.5
22	0.031913	0.564465	0.883688	Median	Range	21.5	22.5
23	0.029653	0.628838	0.883688	Median	Range	22.5	23.5
24	0.026134	0.706951	0.883688	Median	Range	23.5	24.5
5	-0.07208	0.237283	0.316378	Max	Range	4.5	5.5
6	-0.07951	0.127031	0.195432	Max	Range	5.5	6.5
7	-0.0855	0.059302	0.118603	Max	Range	6.5	7.5
8	-0.09004	0.026987	0.077104	Max	Range	7.5	8.5
9	-0.09313	0.01443	0.065009	Max	Range	8.5	9.5
10	-0.09477	0.01043	0.065009	Max	Range	9.5	10.5
11	-0.09496	0.010178	0.065009	Max	Range	10.5	11.5
12	-0.09371	0.012188	0.065009	Max	Range	11.5	12.5
13	-0.09101	0.016252	0.065009	Max	Range	12.5	13.5
14	-0.08686	0.022763	0.075875	Max	Range	13.5	14.5
15	-0.08126	0.032777	0.081942	Max	Range	14.5	15.5
16	-0.07422	0.048673	0.108162	Max	Range	15.5	16.5
17	-0.06572	0.075766	0.137756	Max	Range	16.5	17.5
18	-0.05578	0.12576	0.195432	Max	Range	17.5	18.5
19	-0.04439	0.221975	0.316378	Max	Range	18.5	19.5
20	-0.03156	0.397771	0.497213	Max	Range	19.5	20.5
21	-0.01727	0.665218	0.739131	Max	Range	20.5	21.5

22	-0.00154	0.972421	0.972421	Max	Range	21.5	22.5
23	0.01564	0.760866	0.800912	Max	Range	22.5	23.5
24	0.034269	0.570184	0.670804	Max	Range	23.5	24.5
5	0.010699	0.792348	0.880387	Variability	Range	4.5	5.5
6	-0.00017	0.996026	0.996026	Variability	Range	5.5	6.5
7	-0.00992	0.731616	0.860725	Variability	Range	6.5	7.5
8	-0.01856	0.464881	0.619841	Variability	Range	7.5	8.5
9	-0.02608	0.26461	0.407093	Variability	Range	8.5	9.5
10	-0.03249	0.150743	0.274078	Variability	Range	9.5	10.5
11	-0.03778	0.095316	0.211813	Variability	Range	10.5	11.5
12	-0.04196	0.068794	0.184023	Variability	Range	11.5	12.5
13	-0.04501	0.055262	0.184023	Variability	Range	12.5	13.5
14	-0.04696	0.04777	0.184023	Variability	Range	13.5	14.5
15	-0.04779	0.043546	0.184023	Variability	Range	14.5	15.5
16	-0.0475	0.041867	0.184023	Variability	Range	15.5	16.5
17	-0.0461	0.043514	0.184023	Variability	Range	16.5	17.5
18	-0.04358	0.051466	0.184023	Variability	Range	17.5	18.5
19	-0.03995	0.073609	0.184023	Variability	Range	18.5	19.5
20	-0.0352	0.128437	0.256873	Variability	Range	19.5	20.5
21	-0.02933	0.245972	0.407093	Variability	Range	20.5	21.5
22	-0.02235	0.440458	0.619841	Variability	Range	21.5	22.5
23	-0.01426	0.677309	0.846637	Variability	Range	22.5	23.5
24	-0.00504	0.902135	0.949616	Variability	Range	23.5	24.5
5	-0.19049	0.016342	0.267285	Mean	Angular.Velocity	4.5	5.5
6	-0.15256	0.026729	0.267285	Mean	Angular.Velocity	5.5	6.5
7	-0.11836	0.052231	0.348207	Mean	Angular.Velocity	6.5	7.5
8	-0.08791	0.114149	0.570743	Mean	Angular.Velocity	7.5	8.5
9	-0.0612	0.244763	0.699322	Mean	Angular.Velocity	8.5	9.5
10	-0.03822	0.456803	0.971513	Mean	Angular.Velocity	9.5	10.5
11	-0.01899	0.711159	0.971513	Mean	Angular.Velocity	10.5	11.5
12	-0.00349	0.94622	0.995426	Mean	Angular.Velocity	11.5	12.5
13	0.008268	0.874362	0.971513	Mean	Angular.Velocity	12.5	13.5
14	0.016286	0.756945	0.971513	Mean	Angular.Velocity	13.5	14.5
15	0.020565	0.695743	0.971513	Mean	Angular.Velocity	14.5	15.5
16	0.021105	0.685834	0.971513	Mean	Angular.Velocity	15.5	16.5
17	0.017906	0.728161	0.971513	Mean	Angular.Velocity	16.5	17.5
18	0.010968	0.829408	0.971513	Mean	Angular.Velocity	17.5	18.5
19	0.000291	0.995426	0.995426	Mean	Angular.Velocity	18.5	19.5
20	-0.01413	0.785001	0.971513	Mean	Angular.Velocity	19.5	20.5
21	-0.03228	0.553699	0.971513	Mean	Angular.Velocity	20.5	21.5
22	-0.05418	0.362736	0.906841	Mean	Angular.Velocity	21.5	22.5
23	-0.07981	0.234387	0.699322	Mean	Angular.Velocity	22.5	23.5
24	-0.10918	0.157952	0.631809	Mean	Angular.Velocity	23.5	24.5
5	-0.13387	0.08883	0.962846	Median	Angular.Velocity	4.5	5.5
6	-0.11027	0.109742	0.962846	Median	Angular.Velocity	5.5	6.5
7	-0.08849	0.151505	0.962846	Median	Angular.Velocity	6.5	7.5

8	-0.06852	0.228091	0.962846	Median	Angular.Velocity	7.5	8.5
9	-0.05037	0.351945	0.962846	Median	Angular.Velocity	8.5	9.5
10	-0.03405	0.520674	0.962846	Median	Angular.Velocity	9.5	10.5
11	-0.01954	0.712059	0.962846	Median	Angular.Velocity	10.5	11.5
12	-0.00685	0.8979	0.962846	Median	Angular.Velocity	11.5	12.5
13	0.004025	0.940423	0.962846	Median	Angular.Velocity	12.5	13.5
14	0.013078	0.809208	0.962846	Median	Angular.Velocity	13.5	14.5
15	0.020312	0.707521	0.962846	Median	Angular.Velocity	14.5	15.5
16	0.025728	0.632315	0.962846	Median	Angular.Velocity	15.5	16.5
17	0.029324	0.581352	0.962846	Median	Angular.Velocity	16.5	17.5
18	0.031102	0.554525	0.962846	Median	Angular.Velocity	17.5	18.5
19	0.031061	0.554207	0.962846	Median	Angular.Velocity	18.5	19.5
20	0.029201	0.584362	0.962846	Median	Angular.Velocity	19.5	20.5
21	0.025523	0.647513	0.962846	Median	Angular.Velocity	20.5	21.5
22	0.020026	0.740038	0.962846	Median	Angular.Velocity	21.5	22.5
23	0.01271	0.850205	0.962846	Median	Angular.Velocity	22.5	23.5
24	0.003575	0.962846	0.962846	Median	Angular.Velocity	23.5	24.5
5	-0.13441	0.057894	0.180954	Max	Angular.Velocity	4.5	5.5
6	-0.12337	0.038923	0.155694	Max	Angular.Velocity	5.5	6.5
7	-0.11281	0.027451	0.155694	Max	Angular.Velocity	6.5	7.5
8	-0.10272	0.023164	0.155694	Max	Angular.Velocity	7.5	8.5
9	-0.09311	0.026101	0.155694	Max	Angular.Velocity	8.5	9.5
10	-0.08396	0.038287	0.155694	Max	Angular.Velocity	9.5	10.5
11	-0.0753	0.063334	0.180954	Max	Angular.Velocity	10.5	11.5
12	-0.0671	0.103344	0.25836	Max	Angular.Velocity	11.5	12.5
13	-0.05938	0.156435	0.347633	Max	Angular.Velocity	12.5	13.5
14	-0.05213	0.217903	0.435805	Max	Angular.Velocity	13.5	14.5
15	-0.04536	0.283123	0.514768	Max	Angular.Velocity	14.5	15.5
16	-0.03906	0.349475	0.582459	Max	Angular.Velocity	15.5	16.5
17	-0.03323	0.416956	0.641471	Max	Angular.Velocity	16.5	17.5
18	-0.02787	0.488008	0.697154	Max	Angular.Velocity	17.5	18.5
19	-0.02299	0.566293	0.755057	Max	Angular.Velocity	18.5	19.5
20	-0.01859	0.653224	0.81653	Max	Angular.Velocity	19.5	20.5
21	-0.01465	0.743361	0.874542	Max	Angular.Velocity	20.5	21.5
22	-0.01119	0.825342	0.917047	Max	Angular.Velocity	21.5	22.5
23	-0.00821	0.890074	0.935712	Max	Angular.Velocity	22.5	23.5
24	-0.00569	0.935712	0.935712	Max	Angular.Velocity	23.5	24.5
5	0.01075	0.819363	0.933187	Variability	Angular.Velocity	4.5	5.5
6	0.001085	0.97813	0.97813	Variability	Angular.Velocity	5.5	6.5
7	-0.00746	0.825224	0.933187	Variability	Angular.Velocity	6.5	7.5
8	-0.01489	0.617687	0.933187	Variability	Angular.Velocity	7.5	8.5
9	-0.02119	0.442505	0.804554	Variability	Angular.Velocity	8.5	9.5
10	-0.02638	0.324346	0.72077	Variability	Angular.Velocity	9.5	10.5
11	-0.03044	0.256537	0.72077	Variability	Angular.Velocity	10.5	11.5
12	-0.03338	0.221277	0.72077	Variability	Angular.Velocity	11.5	12.5
13	-0.03521	0.204962	0.72077	Variability	Angular.Velocity	12.5	13.5

14	-0.03591	0.2003	0.72077	Variability	Angular.Velocity	13.5	14.5
15	-0.03549	0.204582	0.72077	Variability	Angular.Velocity	14.5	15.5
16	-0.03396	0.218602	0.72077	Variability	Angular.Velocity	15.5	16.5
17	-0.0313	0.246982	0.72077	Variability	Angular.Velocity	16.5	17.5
18	-0.02752	0.299813	0.72077	Variability	Angular.Velocity	17.5	18.5
19	-0.02262	0.393949	0.787897	Variability	Angular.Velocity	18.5	19.5
20	-0.0166	0.546559	0.910931	Variability	Angular.Velocity	19.5	20.5
21	-0.00947	0.752698	0.933187	Variability	Angular.Velocity	20.5	21.5
22	-0.00121	0.971975	0.97813	Variability	Angular.Velocity	21.5	22.5
23	0.008173	0.839868	0.933187	Variability	Angular.Velocity	22.5	23.5
24	0.018673	0.698956	0.933187	Variability	Angular.Velocity	23.5	24.5
5	-0.22092	0.018888	0.377751	Mean	Angular.Acceleration	4.5	5.5
6	-0.16619	0.042277	0.422769	Mean	Angular.Acceleration	5.5	6.5
7	-0.11683	0.107612	0.523957	Mean	Angular.Acceleration	6.5	7.5
8	-0.07286	0.272449	0.523957	Mean	Angular.Acceleration	7.5	8.5
9	-0.03426	0.585635	0.732044	Mean	Angular.Acceleration	8.5	9.5
10	-0.00105	0.986398	0.986398	Mean	Angular.Acceleration	9.5	10.5
11	0.02679	0.661949	0.778764	Mean	Angular.Acceleration	10.5	11.5
12	0.049248	0.425569	0.654721	Mean	Angular.Acceleration	11.5	12.5
13	0.066326	0.288176	0.523957	Mean	Angular.Acceleration	12.5	13.5
14	0.078024	0.214336	0.523957	Mean	Angular.Acceleration	13.5	14.5
15	0.084344	0.179244	0.523957	Mean	Angular.Acceleration	14.5	15.5
16	0.085283	0.1712	0.523957	Mean	Angular.Acceleration	15.5	16.5
17	0.080843	0.189186	0.523957	Mean	Angular.Acceleration	16.5	17.5
18	0.071024	0.243319	0.523957	Mean	Angular.Acceleration	17.5	18.5
19	0.055825	0.358211	0.597019	Mean	Angular.Acceleration	18.5	19.5
20	0.035247	0.569229	0.732044	Mean	Angular.Acceleration	19.5	20.5
21	0.009289	0.886579	0.933241	Mean	Angular.Acceleration	20.5	21.5
22	-0.02205	0.756116	0.840129	Mean	Angular.Acceleration	21.5	22.5
23	-0.05877	0.461906	0.659866	Mean	Angular.Acceleration	22.5	23.5
24	-0.10086	0.272093	0.523957	Mean	Angular.Acceleration	23.5	24.5
5	-0.13051	0.102426	0.941738	Median	Angular.Acceleration	4.5	5.5
6	-0.10315	0.140917	0.941738	Median	Angular.Acceleration	5.5	6.5
7	-0.07813	0.212808	0.941738	Median	Angular.Acceleration	6.5	7.5
8	-0.05544	0.337411	0.941738	Median	Angular.Acceleration	7.5	8.5
9	-0.03508	0.523612	0.941738	Median	Angular.Acceleration	8.5	9.5
10	-0.01707	0.751388	0.97093	Median	Angular.Acceleration	9.5	10.5
11	-0.00138	0.979482	0.979482	Median	Angular.Acceleration	10.5	11.5
12	0.011964	0.825291	0.97093	Median	Angular.Acceleration	11.5	12.5
13	0.022975	0.674474	0.963535	Median	Angular.Acceleration	12.5	13.5
14	0.031651	0.565043	0.941738	Median	Angular.Acceleration	13.5	14.5
15	0.037991	0.489609	0.941738	Median	Angular.Acceleration	14.5	15.5
16	0.041995	0.441953	0.941738	Median	Angular.Acceleration	15.5	16.5
17	0.043665	0.418985	0.941738	Median	Angular.Acceleration	16.5	17.5
18	0.042998	0.421361	0.941738	Median	Angular.Acceleration	17.5	18.5
19	0.039996	0.453611	0.941738	Median	Angular.Acceleration	18.5	19.5

20	0.034659	0.522912	0.941738	Median	Angular.Acceleration	19.5	20.5
21	0.026986	0.634213	0.963535	Median	Angular.Acceleration	20.5	21.5
22	0.016977	0.781784	0.97093	Median	Angular.Acceleration	21.5	22.5
23	0.004633	0.945915	0.979482	Median	Angular.Acceleration	22.5	23.5
24	-0.01005	0.897313	0.979482	Median	Angular.Acceleration	23.5	24.5
5	-0.16117	0.095891	0.82945	Max	Angular.Acceleration	4.5	5.5
6	-0.13179	0.102454	0.82945	Max	Angular.Acceleration	5.5	6.5
7	-0.10466	0.124418	0.82945	Max	Angular.Acceleration	6.5	7.5
8	-0.07979	0.178429	0.850577	Max	Angular.Acceleration	7.5	8.5
9	-0.05716	0.291297	0.850577	Max	Angular.Acceleration	8.5	9.5
10	-0.03679	0.480461	0.850577	Max	Angular.Acceleration	9.5	10.5
11	-0.01867	0.720528	0.90066	Max	Angular.Acceleration	10.5	11.5
12	-0.0028	0.958076	0.958076	Max	Angular.Acceleration	11.5	12.5
13	0.010825	0.841934	0.935483	Max	Angular.Acceleration	12.5	13.5
14	0.022195	0.686287	0.90066	Max	Angular.Acceleration	13.5	14.5
15	0.031314	0.568642	0.874835	Max	Angular.Acceleration	14.5	15.5
16	0.038182	0.481307	0.850577	Max	Angular.Acceleration	15.5	16.5
17	0.042799	0.419926	0.850577	Max	Angular.Acceleration	16.5	17.5
18	0.045165	0.385084	0.850577	Max	Angular.Acceleration	17.5	18.5
19	0.045281	0.382827	0.850577	Max	Angular.Acceleration	18.5	19.5
20	0.043145	0.422821	0.850577	Max	Angular.Acceleration	19.5	20.5
21	0.038759	0.510346	0.850577	Max	Angular.Acceleration	20.5	21.5
22	0.032121	0.634868	0.90066	Max	Angular.Acceleration	21.5	22.5
23	0.023233	0.771998	0.908232	Max	Angular.Acceleration	22.5	23.5
24	0.012094	0.899997	0.947365	Max	Angular.Acceleration	23.5	24.5
5	0.010768	0.80777	0.980156	Variability	Angular.Acceleration	4.5	5.5
6	0.004594	0.901539	0.980156	Variability	Angular.Acceleration	5.5	6.5
7	-0.00079	0.980156	0.980156	Variability	Angular.Acceleration	6.5	7.5
8	-0.00537	0.847203	0.980156	Variability	Angular.Acceleration	7.5	8.5
9	-0.00917	0.722187	0.980156	Variability	Angular.Acceleration	8.5	9.5
10	-0.01217	0.626644	0.980156	Variability	Angular.Acceleration	9.5	10.5
11	-0.01438	0.566741	0.980156	Variability	Angular.Acceleration	10.5	11.5
12	-0.01579	0.536548	0.980156	Variability	Angular.Acceleration	11.5	12.5
13	-0.01641	0.528096	0.980156	Variability	Angular.Acceleration	12.5	13.5
14	-0.01623	0.536318	0.980156	Variability	Angular.Acceleration	13.5	14.5
15	-0.01527	0.559841	0.980156	Variability	Angular.Acceleration	14.5	15.5
16	-0.0135	0.600782	0.980156	Variability	Angular.Acceleration	15.5	16.5
17	-0.01095	0.664599	0.980156	Variability	Angular.Acceleration	16.5	17.5
18	-0.0076	0.759013	0.980156	Variability	Angular.Acceleration	17.5	18.5
19	-0.00346	0.888965	0.980156	Variability	Angular.Acceleration	18.5	19.5
20	0.001476	0.954304	0.980156	Variability	Angular.Acceleration	19.5	20.5
21	0.007205	0.798262	0.980156	Variability	Angular.Acceleration	20.5	21.5
22	0.013728	0.671157	0.980156	Variability	Angular.Acceleration	21.5	22.5
23	0.021045	0.581877	0.980156	Variability	Angular.Acceleration	22.5	23.5
24	0.029155	0.523713	0.980156	Variability	Angular.Acceleration	23.5	24.5
5	0.009582	0.424077	0.738018	Mean	Entropy	4.5	5.5

6	0.006309	0.569425	0.759233	Mean	Entropy	5.5	6.5
7	0.003312	0.751464	0.83496	Mean	Entropy	6.5	7.5
8	0.000592	0.953041	0.953041	Mean	Entropy	7.5	8.5
9	-0.00185	0.850884	0.895667	Mean	Entropy	8.5	9.5
10	-0.00402	0.680722	0.80085	Mean	Entropy	9.5	10.5
11	-0.0059	0.545109	0.759233	Mean	Entropy	10.5	11.5
12	-0.00752	0.442811	0.738018	Mean	Entropy	11.5	12.5
13	-0.00885	0.368121	0.738018	Mean	Entropy	12.5	13.5
14	-0.00991	0.314843	0.738018	Mean	Entropy	13.5	14.5
15	-0.01069	0.278052	0.738018	Mean	Entropy	14.5	15.5
16	-0.01119	0.254524	0.738018	Mean	Entropy	15.5	16.5
17	-0.01142	0.242706	0.738018	Mean	Entropy	16.5	17.5
18	-0.01137	0.242671	0.738018	Mean	Entropy	17.5	18.5
19	-0.01104	0.256183	0.738018	Mean	Entropy	18.5	19.5
20	-0.01044	0.286793	0.738018	Mean	Entropy	19.5	20.5
21	-0.00956	0.339513	0.738018	Mean	Entropy	20.5	21.5
22	-0.0084	0.419254	0.738018	Mean	Entropy	21.5	22.5
23	-0.00696	0.527595	0.759233	Mean	Entropy	22.5	23.5
24	-0.00525	0.65948	0.80085	Mean	Entropy	23.5	24.5
5	0.01157	0.402028	0.73096	Median	Entropy	4.5	5.5
6	0.007911	0.533833	0.737156	Median	Entropy	5.5	6.5
7	0.004545	0.703533	0.81403	Median	Entropy	6.5	7.5
8	0.001473	0.897641	0.907208	Median	Entropy	7.5	8.5
9	-0.0013	0.907208	0.907208	Median	Entropy	8.5	9.5
10	-0.00379	0.732627	0.81403	Median	Entropy	9.5	10.5
11	-0.00598	0.589725	0.737156	Median	Entropy	10.5	11.5
12	-0.00787	0.479291	0.737156	Median	Entropy	11.5	12.5
13	-0.00948	0.396675	0.73096	Median	Entropy	12.5	13.5
14	-0.01078	0.336018	0.73096	Median	Entropy	13.5	14.5
15	-0.0118	0.292348	0.73096	Median	Entropy	14.5	15.5
16	-0.01252	0.262225	0.73096	Median	Entropy	15.5	16.5
17	-0.01294	0.24378	0.73096	Median	Entropy	16.5	17.5
18	-0.01307	0.236675	0.73096	Median	Entropy	17.5	18.5
19	-0.01291	0.242131	0.73096	Median	Entropy	18.5	19.5
20	-0.01246	0.263017	0.73096	Median	Entropy	19.5	20.5
21	-0.01171	0.303645	0.73096	Median	Entropy	20.5	21.5
22	-0.01066	0.368585	0.73096	Median	Entropy	21.5	22.5
23	-0.00932	0.459996	0.737156	Median	Entropy	22.5	23.5
24	-0.00769	0.574577	0.737156	Median	Entropy	23.5	24.5
5	-0.0004	0.956881	0.997209	Max	Entropy	4.5	5.5
6	-0.00048	0.93849	0.997209	Max	Entropy	5.5	6.5
7	-0.00053	0.923616	0.997209	Max	Entropy	6.5	7.5
8	-0.00052	0.916345	0.997209	Max	Entropy	7.5	8.5
9	-0.00046	0.920182	0.997209	Max	Entropy	8.5	9.5
10	-0.00036	0.936059	0.997209	Max	Entropy	9.5	10.5
11	-0.00021	0.962432	0.997209	Max	Entropy	10.5	11.5

12	-1.60E-05	0.997209	0.997209	Max	Entropy	11.5	12.5
13	0.000228	0.960772	0.997209	Max	Entropy	12.5	13.5
14	0.000519	0.911476	0.997209	Max	Entropy	13.5	14.5
15	0.000858	0.85406	0.997209	Max	Entropy	14.5	15.5
16	0.001244	0.787535	0.997209	Max	Entropy	15.5	16.5
17	0.001678	0.711817	0.997209	Max	Entropy	16.5	17.5
18	0.002158	0.629388	0.997209	Max	Entropy	17.5	18.5
19	0.002687	0.547114	0.997209	Max	Entropy	18.5	19.5
20	0.003262	0.476018	0.997209	Max	Entropy	19.5	20.5
21	0.003885	0.426581	0.997209	Max	Entropy	20.5	21.5
22	0.004556	0.402479	0.997209	Max	Entropy	21.5	22.5
23	0.005273	0.399635	0.997209	Max	Entropy	22.5	23.5
24	0.006038	0.41061	0.997209	Max	Entropy	23.5	24.5
5	-0.0168	0.427126	0.502501	Variability	Entropy	4.5	5.5
6	-0.00486	0.801427	0.801427	Variability	Entropy	5.5	6.5
7	0.005901	0.743542	0.782676	Variability	Entropy	6.5	7.5
8	0.015492	0.367628	0.459535	Variability	Entropy	7.5	8.5
9	0.023911	0.152984	0.218549	Variability	Entropy	8.5	9.5
10	0.031157	0.059619	0.099365	Variability	Entropy	9.5	10.5
11	0.037231	0.02423	0.04846	Variability	Entropy	10.5	11.5
12	0.042132	0.01107	0.027674	Variability	Entropy	11.5	12.5
13	0.04586	0.005914	0.019713	Variability	Entropy	12.5	13.5
14	0.048416	0.003759	0.017727	Variability	Entropy	13.5	14.5
15	0.0498	0.002869	0.017727	Variability	Entropy	14.5	15.5
16	0.05001	0.002662	0.017727	Variability	Entropy	15.5	16.5
17	0.049048	0.003056	0.017727	Variability	Entropy	16.5	17.5
18	0.046914	0.004432	0.017727	Variability	Entropy	17.5	18.5
19	0.043606	0.008201	0.023432	Variability	Entropy	18.5	19.5
20	0.039126	0.01895	0.042112	Variability	Entropy	19.5	20.5
21	0.033474	0.050662	0.092113	Variability	Entropy	20.5	21.5
22	0.026649	0.13785	0.212077	Variability	Entropy	21.5	22.5
23	0.018651	0.332701	0.443601	Variability	Entropy	22.5	23.5
24	0.009481	0.652694	0.725215	Variability	Entropy	23.5	24.5
5	-0.08961	0.446236	0.957926	Mean	Jerk	4.5	5.5
6	-0.07314	0.481838	0.957926	Mean	Jerk	5.5	6.5
7	-0.05769	0.538778	0.957926	Mean	Jerk	6.5	7.5
8	-0.04325	0.619434	0.957926	Mean	Jerk	7.5	8.5
9	-0.02983	0.720081	0.957926	Mean	Jerk	8.5	9.5
10	-0.01743	0.830961	0.957926	Mean	Jerk	9.5	10.5
11	-0.00604	0.940891	0.957926	Mean	Jerk	10.5	11.5
12	0.004327	0.957926	0.957926	Mean	Jerk	11.5	12.5
13	0.01368	0.868647	0.957926	Mean	Jerk	12.5	13.5
14	0.022015	0.791239	0.957926	Mean	Jerk	13.5	14.5
15	0.029333	0.724366	0.957926	Mean	Jerk	14.5	15.5
16	0.035634	0.666709	0.957926	Mean	Jerk	15.5	16.5
17	0.040918	0.617746	0.957926	Mean	Jerk	16.5	17.5

18	0.045186	0.578219	0.957926	Mean	Jerk	17.5	18.5
19	0.048436	0.550355	0.957926	Mean	Jerk	18.5	19.5
20	0.050669	0.537559	0.957926	Mean	Jerk	19.5	20.5
21	0.051885	0.543145	0.957926	Mean	Jerk	20.5	21.5
22	0.052084	0.568218	0.957926	Mean	Jerk	21.5	22.5
23	0.051267	0.610043	0.957926	Mean	Jerk	22.5	23.5
24	0.049432	0.662631	0.957926	Mean	Jerk	23.5	24.5
5	0.0075	0.948436	0.973089	Median	Jerk	4.5	5.5
6	-0.00347	0.973089	0.973089	Median	Jerk	5.5	6.5
7	-0.01279	0.890961	0.973089	Median	Jerk	6.5	7.5
8	-0.02045	0.813773	0.973089	Median	Jerk	7.5	8.5
9	-0.02645	0.750197	0.973089	Median	Jerk	8.5	9.5
10	-0.0308	0.705401	0.973089	Median	Jerk	9.5	10.5
11	-0.03348	0.680152	0.973089	Median	Jerk	10.5	11.5
12	-0.03451	0.672613	0.973089	Median	Jerk	11.5	12.5
13	-0.03389	0.680518	0.973089	Median	Jerk	12.5	13.5
14	-0.0316	0.702443	0.973089	Median	Jerk	13.5	14.5
15	-0.02766	0.738213	0.973089	Median	Jerk	14.5	15.5
16	-0.02206	0.788857	0.973089	Median	Jerk	15.5	16.5
17	-0.01481	0.856219	0.973089	Median	Jerk	16.5	17.5
18	-0.0059	0.94203	0.973089	Median	Jerk	17.5	18.5
19	0.004674	0.953934	0.973089	Median	Jerk	18.5	19.5
20	0.016901	0.836429	0.973089	Median	Jerk	19.5	20.5
21	0.030785	0.716141	0.973089	Median	Jerk	20.5	21.5
22	0.046326	0.606829	0.973089	Median	Jerk	21.5	22.5
23	0.063524	0.518814	0.973089	Median	Jerk	22.5	23.5
24	0.082379	0.454853	0.973089	Median	Jerk	23.5	24.5
5	-0.00553	0.972102	0.978468	Max	Jerk	4.5	5.5
6	-0.01834	0.893473	0.978468	Max	Jerk	5.5	6.5
7	-0.02928	0.808454	0.978468	Max	Jerk	6.5	7.5
8	-0.03837	0.726839	0.978468	Max	Jerk	7.5	8.5
9	-0.04561	0.659764	0.978468	Max	Jerk	8.5	9.5
10	-0.05099	0.613808	0.978468	Max	Jerk	9.5	10.5
11	-0.05451	0.588906	0.978468	Max	Jerk	10.5	11.5
12	-0.05618	0.581246	0.978468	Max	Jerk	11.5	12.5
13	-0.05599	0.586808	0.978468	Max	Jerk	12.5	13.5
14	-0.05395	0.603069	0.978468	Max	Jerk	13.5	14.5
15	-0.05005	0.629352	0.978468	Max	Jerk	14.5	15.5
16	-0.0443	0.666701	0.978468	Max	Jerk	15.5	16.5
17	-0.03669	0.71766	0.978468	Max	Jerk	16.5	17.5
18	-0.02723	0.785656	0.978468	Max	Jerk	17.5	18.5
19	-0.01591	0.873232	0.978468	Max	Jerk	18.5	19.5
20	-0.00274	0.978468	0.978468	Max	Jerk	19.5	20.5
21	0.012289	0.9084	0.978468	Max	Jerk	20.5	21.5
22	0.029173	0.80264	0.978468	Max	Jerk	21.5	22.5
23	0.047913	0.71634	0.978468	Max	Jerk	22.5	23.5

24	0.068507	0.652817	0.978468	Max	Jerk	23.5	24.5
5	-0.00856	0.865606	0.975817	Variability	Jerk	4.5	5.5
6	-0.00393	0.927026	0.975817	Variability	Jerk	5.5	6.5
7	0.000302	0.993488	0.993488	Variability	Jerk	6.5	7.5
8	0.004128	0.900382	0.975817	Variability	Jerk	7.5	8.5
9	0.007551	0.805802	0.975817	Variability	Jerk	8.5	9.5
10	0.010571	0.723293	0.975817	Variability	Jerk	9.5	10.5
11	0.013188	0.659195	0.975817	Variability	Jerk	10.5	11.5
12	0.015401	0.611959	0.975817	Variability	Jerk	11.5	12.5
13	0.017211	0.576922	0.975817	Variability	Jerk	12.5	13.5
14	0.018617	0.54983	0.975817	Variability	Jerk	13.5	14.5
15	0.019621	0.527998	0.975817	Variability	Jerk	14.5	15.5
16	0.020221	0.510487	0.975817	Variability	Jerk	15.5	16.5
17	0.020418	0.498286	0.975817	Variability	Jerk	16.5	17.5
18	0.020211	0.49477	0.975817	Variability	Jerk	17.5	18.5
19	0.019602	0.505942	0.975817	Variability	Jerk	18.5	19.5
20	0.018589	0.53889	0.975817	Variability	Jerk	19.5	20.5
21	0.017173	0.596799	0.975817	Variability	Jerk	20.5	21.5
22	0.015353	0.673673	0.975817	Variability	Jerk	21.5	22.5
23	0.013131	0.75654	0.975817	Variability	Jerk	22.5	23.5
24	0.010505	0.833829	0.975817	Variability	Jerk	23.5	24.5

S 6

Sensitivity Analysis

Table S10. Sensitivity analysis utilising a random slope for age.

Variable	Effect	Coefficient	SE	Z Value	sig
**Entropy Variability**	**Group**	**0.045**	**0.017**	**2.264**	**0.008**
	**Age (Week): Quadratic**	0.0005	0.0001	**4.502**	**<.0001**
	**Age (Week): Linear**	0.002	0.0007	3.592	<.001
	**Gestational Age**	-0.002	0.0045	-0.509	0.61
	**Group × Age (Week): Quadratic**	-0.0006	0.0001	-3.967	<.001
	**Group × Age (Week): Linear**	0.0009	0.001	0.931	0.351
